# Supplementary material for: Modulation of SF1 Neuron Activity Coordinately Regulates Both Feeding Behavior and Associated Emotional States
Source: Cell Rep. 2017 Dec 19;21(12):3559–72. doi: 10.1016/j.celrep.2017.11.089 (PMC5746599; doi:10.1016/j.celrep.2017.11.089)
Supplement: Document S1. Supplemental Experimental Procedures and Figures S1–S6 [file mmc1.pdf]

Cell Reports, Volume *21*

Supplemental Information

Modulation of SF1 Neuron Activity

Coordinately Regulates Both Feeding Behavior

and Associated Emotional States

Paulius Viskaitis, Elaine E. Irvine, Mark A. Smith, Agharul I. Choudhury, Elisa Alvarez-Curto, Justyna A. Glegola, Darran G. Hardy, Silvia M.A. Pedroni, Maria R. Paiva Pessoa, Anushka B.P. Fernando, Loukia Katsouri, Alessandro Sardini, Mark A. Ungless, Graeme Milligan, and Dominic J. Withers

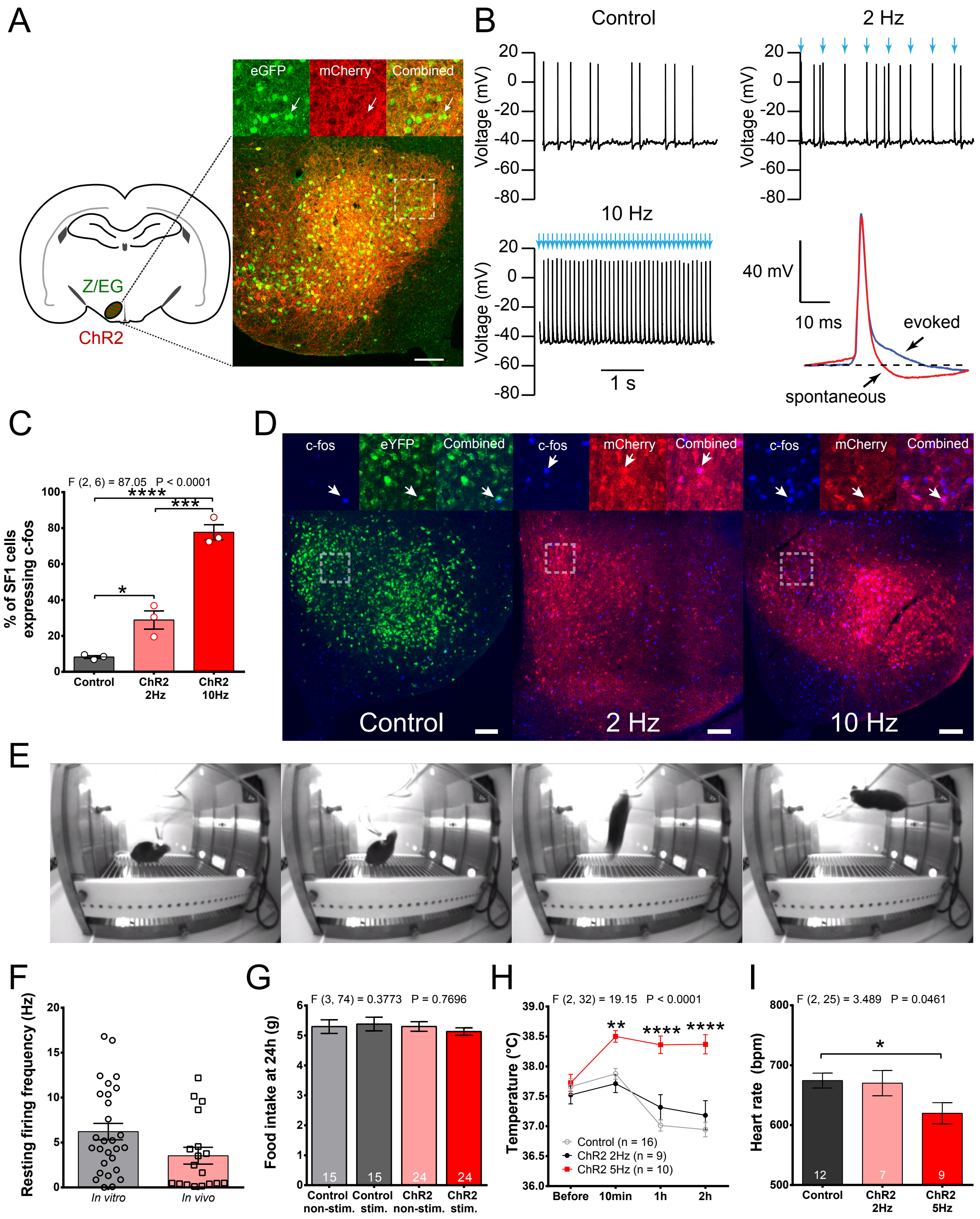

A
eGFP
mCherry
Combined
Z/EG
ChR2
B
Control
2 Hz
Voltage (mV)
20
0
-20
-40
-60
-80
10 Hz
1 s
40 mV
10 ms
evoked
spontaneous
C
F (2, 6) = 87.05 P < 0.0001
% of SF1 cells expressing c-fos
****
***
*
Control
ChR2 2Hz
ChR2 10Hz
D
c-fos
eYFP
Combined
c-fos
mCherry
Combined
c-fos
mCherry
Combined
Control
2 Hz
10 Hz
E
F
Resting firing frequency (Hz)
In vitro
In vivo
G
F (3, 74) = 0.3773 P = 0.7696
Food intake at 24h (g)
15
15
24
24
Control non-stim.
Control stim.
ChR2 non-stim.
ChR2 stim.
H
F (2, 32) = 19.15 P < 0.0001
Temperature (°C)
**
****
****
Control (n = 16)
ChR2 2Hz (n = 9)
ChR2 5Hz (n = 10)
Before
10min
1h
2h
I
F (2, 25) = 3.489 P = 0.0461
Heart rate (bpm)
*
12
7
9
Control
ChR2 2Hz
ChR2 5Hz

Figure S1. Related to Figure 1.
(**A**) Illustration of the anatomic location of the VMH and microscope image showing co-expression of ChR2-mCherry with eGFP in AAV1-DIO-ChR2-mCherry injected SF1-Z/EG mice. Contrast increased for presentation purposes. (**B**) Slice electrophysiology traces during control, 2 Hz and 10 Hz stimulation conditions in current clamp settings, together with representative spontaneous and optogenetically-induced action potentials. (**C**) Quantification of c-fos expression 90 min following the initiation of optogenetic stimulation at 2 or 10 Hz. (**D**) Representative tiled images of the experiment in **C**. Contrast increased uniformly across groups for presentation purposes. (**E**) Example of a 20 Hz frequency optogenetic stimulation-induced escape behaviour in an operant box. (**F**) Steady state firing frequency of SF1 neurons in *ex vivo* (n = 28) or *in vivo* (n = 18) studies. (**G**) 24 h cumulative food intake of SF1-ChR2 mice following 5 h of optogenetic stimulation during a fast-refeed (sample size displayed on the graph). (**H**) Body temperature before or after 10 min, 1 h and 2 h of 2 or 5 Hz optogenetic stimulation. (**I**) Heart rate after continuous 2 h optogenetic stimulation. Scale bars in **A** and **D** are 100 μm. 1-way ANOVA, followed by Sidak post-hoc was performed, with results displayed on all figures except **F** and **H** that were analysed by t-test and 2-way ANOVA respectively. Data presented as mean ± SEM. * $p < 0.05$, ** $p < 0.01$, *** $p < 0.001$, **** $p < 0.0001$.

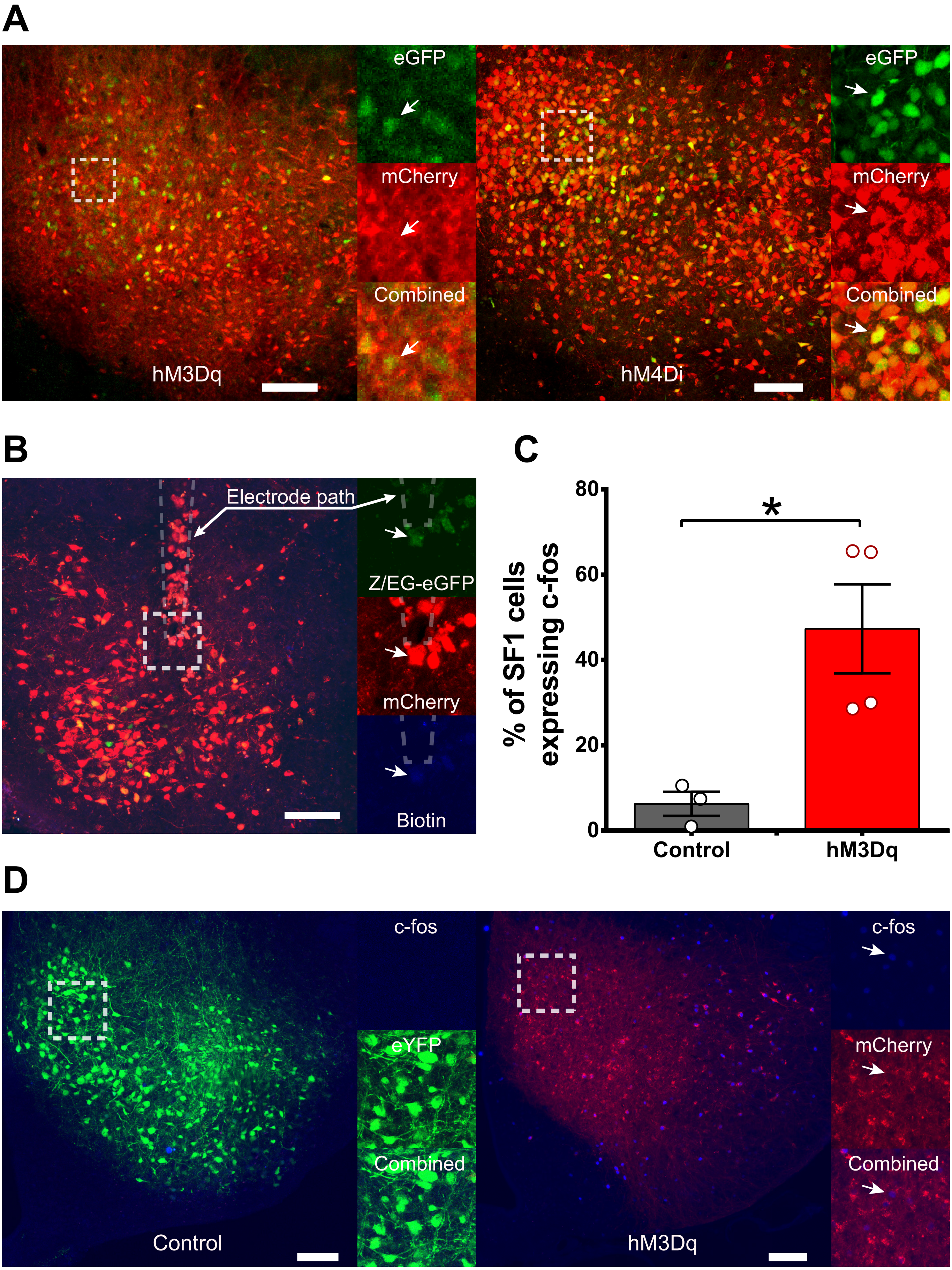
A
eGFP
mCherry
Combined
hM3Dq
eGFP
mCherry
Combined
hM4Di
B
Electrode path
Z/EG-eGFP
mCherry
Biotin
C
*
% of SF1 cells expressing c-fos
80
60
40
20
0
Control
hM3Dq
D
c-fos
eYFP
Combined
Control
c-fos
mCherry
Combined
hM3Dq

Figure S2. Related to Figure 2.
(**A**) Tiled images demonstrating co-expression of mCherry with eGFP in AAV1-DIO-hM3Dq-mCherry or AAV1-DIO-hM4Di-mCherry injected SF1-Z/EG mice. Contrast increased uniformly across groups for representation purposes. (**B**) An example of a resultant electrode path and biotin staining after *in vivo* electrophysiology recordings. (**C**) Proportion of SF1 neurons expressing c-fos (control n = 3; hM3Dq n = 4). (**D**) Representative tiled images of c-fos immunostaining in the VMH of control and SF1-hM3Dq animals 90 min after CNO administration. Contrast increased identically for all images for representation purposes. Scale bars in **A**, **B** and **D** are 100 µm. **C** analysed by a paired t-test. Data presented as mean ± SEM. * $p < 0.05$.

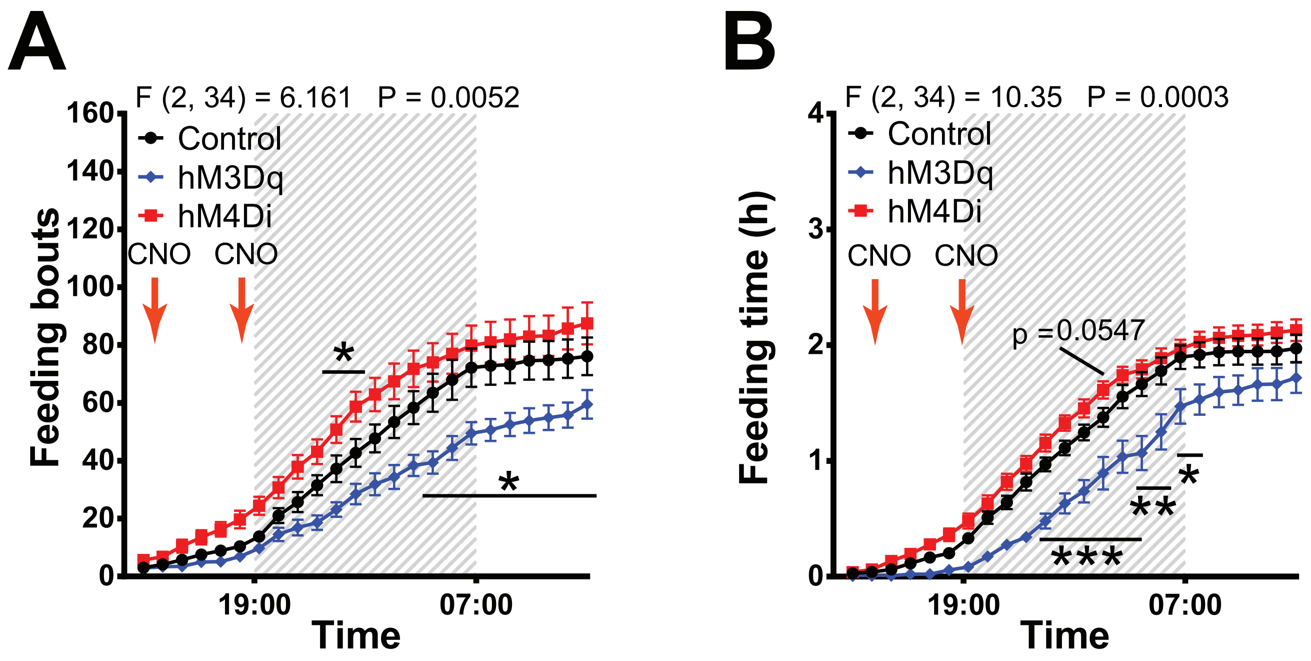

Figure S3. Related to Figure 3.
(**A**) Cumulative numbers of feeding bouts detected in the BioDAQ feeding system (control n = 13, hM3Dq n = 8, hM4Di, n = 16). (**B**) Same experiment as in **A**, but cumulative time spent feeding shown. Analysed by 2-way repeated measures ANOVA, Sidak post-hoc. Data presented as mean ± SEM. * $p < 0.05$, ** $p < 0.01$, *** $p < 0.001$.

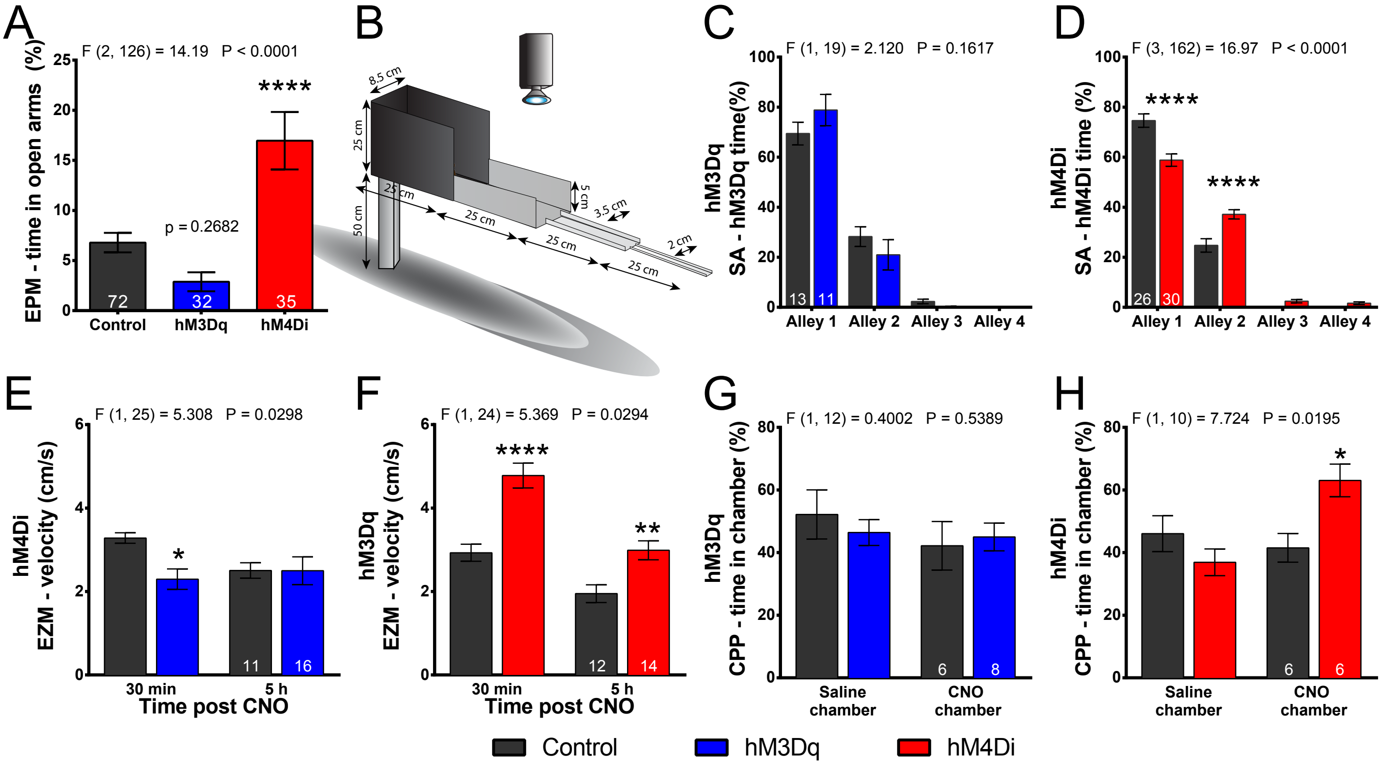

Figure S4. Related to Figure 4.

(**A**) Time spent in open areas of elevated plus maze (EPM). (**B**) Depiction of the successive alleys (SA) arena. Time spent per individual alleys of SA task by SF1-hM3Dq (**C**) and SF1-hM4Di mice (**D**). Velocity of SF1-hM3Dq (**E**) and SF1-hM4Di mice during EZM (**F**). (**G**) Conditioned place preference for chambers in which control or SF1-hM3Dq animals received either vehicle or CNO IP injections. (**H**) Identical to **G**, but results for SF1-hM4Di mice. Sample sizes are displayed on the appropriate columns. **A** analysed by 1-way ANOVA and Sidak post-hoc, while the rest of the studies were analysed by 2-way repeated measures ANOVA, Sidak post-hoc. Data presented as mean ± SEM. * $p < 0.05$, ** $p < 0.01$, **** $p < 0.0001$.

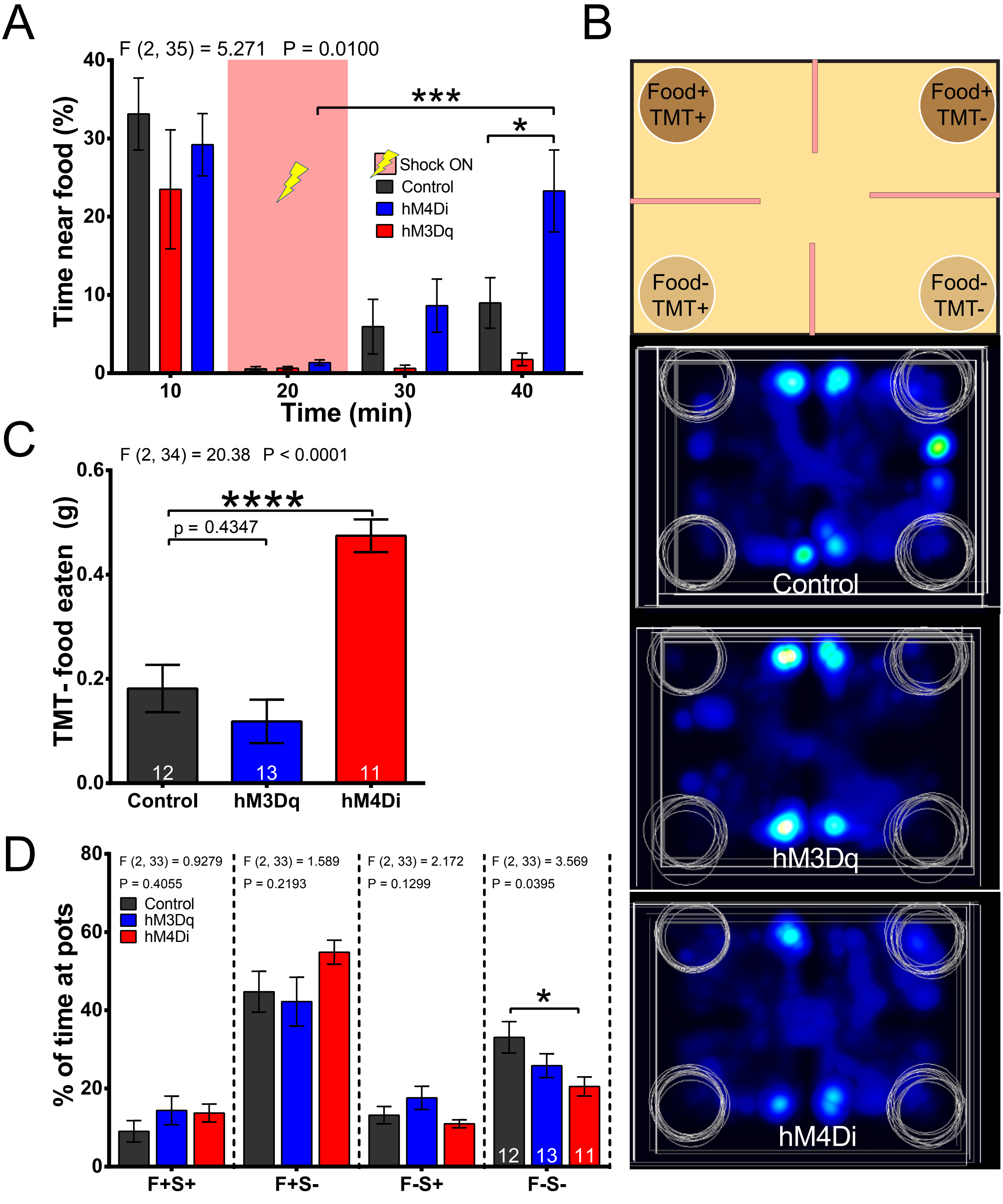

Figure S5. Related to Figure 5.
(**A**) Time spent in the feeding area during the negative feeding conditioning assay (control n = 7; hM3Dq n = 7; hM4Di n = 8). (**B**) Area subdivision of the TMT avoidance assay depicted in Figure 5E and heat-maps of place preference of control, SF1-hM3Dq and SF1-hM4Di mice during this task. (**C**) Food intake from TMT unscented pot. (**D**) Time spent at individual pots divided by the total time spent at all of the pots by that mouse. **A** was analysed by 2-way repeated measures ANOVA, Sidak post-hoc, while **C** and individual pots in **D** were analysed by 1-way ANOVA and Sidak post-hoc tests. Sample sizes indicated on the panels. Data presented as mean ± SEM. * $p < 0.05$, *** $p < 0.001$, **** $p < 0.0001$.

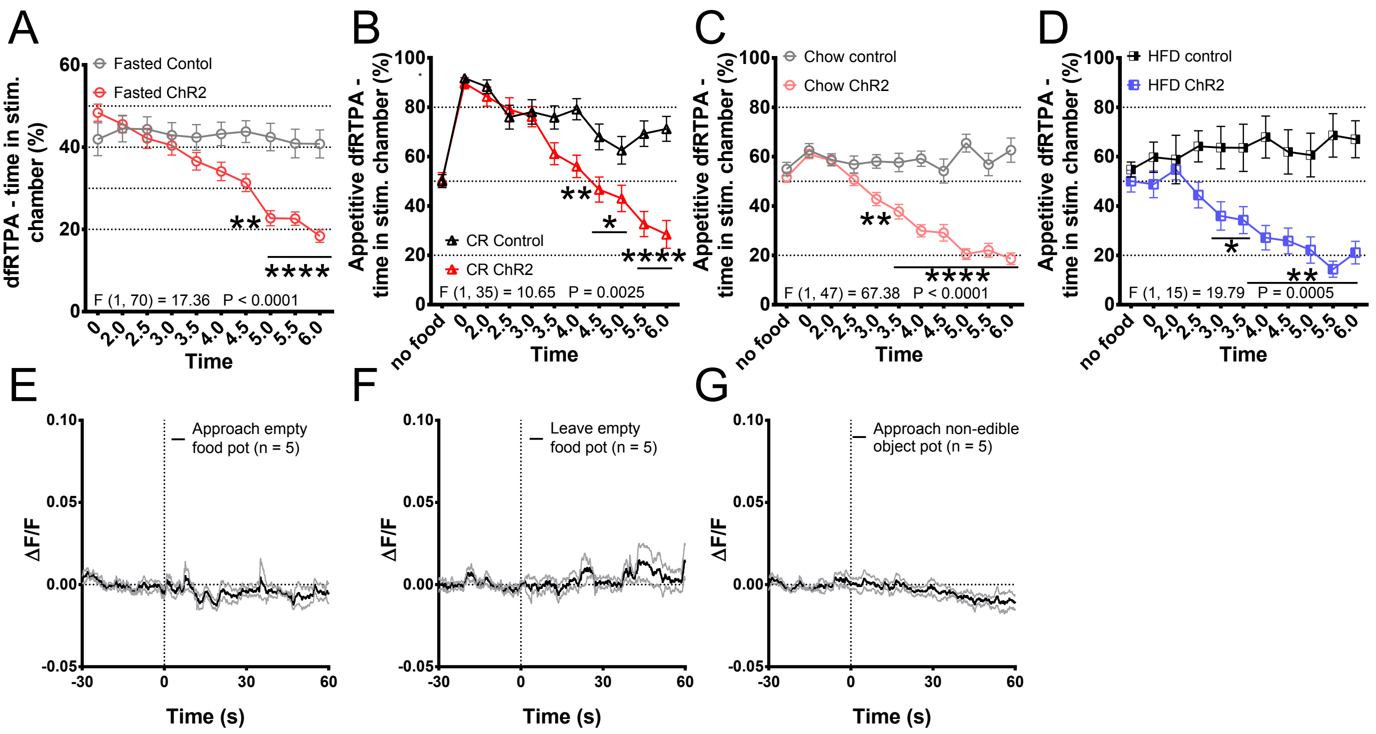

Figure S6. Related to Figure 6.
(**A**) Time spent in stimulation chamber by fasted control and SF1-ChR2 mice during the dfRTPA task (control n = 32; ChR2 = 40). (**B**) Time spent in stimulation chamber by overnight fasted, calorie restricted (CR) control and SF1-ChR2 animals during appetitive dfRTPA assay (control n = 14; ChR2 = 23). (**C**) Same as in **B**, but for chow fed mice (control n = 19; ChR2 = 30). (**D**) Identical to **B** and **C**, but in high-fat fed animals (control n = 8; ChR2 = 9). (**E**) Fluorescence changes in SF1-GCaMP6m mice aligned to approach of an empty pot, later in which food was placed. (**F**-**G**) Fluorescence changes when approaching or leaving an empty food pot, or when approaching a non-edible object. **A**-**D** analysis performed using 2-way repeated measures ANOVA, Sidak post-hoc. 30 s baselines prior to the behaviors were used in **E**-**G**. Data presented as mean ± SEM. * $p < 0.05$, ** $p < 0.01$, **** $p < 0.0001$.

Extended experimental procedures

Key resources table

Reagent or Resource	Source	Identifier
Antibodies		
Anti c-fos	Cell Signaling Technologies	#2250
Anti-rabbit IgG (H+L), conjugated with Alexa Fluor 647	Molecular Probes	A-21443
Experimental Models: Mouse lines		
SF1-Cre	Jackson Laboratory	012462
Z/EG	(Novak et al., 2000)	N/A
Chemicals (Pharmacological compounds)		
Clozapine N-oxide	Key Organics	N/A
Recombinant DNA		
AAV1-Ef1a-DIO-hM3Dq-mCherry	Dr A.I. Choudhury in collaboration with Prof G. Milligan	N/A
AAV1-Ef1a-DIO-hM4Di-mCherry	GeneArt, Thermo Fisher (Armbruster et al., 2007)	N/A
AAV1-Ef1a-DIO-ChR2-mCherry	Prof K. Deisseroth	N/A
AAV1-Ef1a-DIO-EYFP	Addgene	Plasmid #27056
AAV1.Syn.Flex.GCaMP6m.WPRE.SV40	Penn Vector Core	#2820
Sequence-Based Reagents		
NR5A1 forward 5' – CTGAGCTGCAGCGCAGGGACAT – 3'	(Dhillon et al., 2006)	N/A
NR5A1 reverse 5' – TGCGAACCTCATCACTCGTTGCAT – 3'	(Dhillon et al., 2006)	N/A
Z/eg forward 5' – AAGTTCATCTGCACCACCG – 3'	(Novak et al., 2000)	N/A

Z/eg reverse 5' – TCCTTGAAGAAGATGGTGCG – 3'	(Novak et al., 2000)	N/A
Optogenetic and imaging reagents		
Laser CL-473-050	CrystaLaser	CL-473-050
Laser STRADUS-473-80	Laser 2000	473-80
Stimulus generators	Multichannel Systems	STG4004 and STG4008
Beam-splitting, rotary joints	Doric Lenses	FRJ_1x2i_FC-2FC_0.22
Optic cables (200 µm, 0.39 NA, M72L02)	Thorlabs	M72L02
FC/PC to 1.25mm ferrule patch cords	Thorlabs	Custom
Mating sleeves	Thorlabs	AFAF1
nVista microscope	Inscopix	N/A
Baseplate	Inscopix	1050-002192
Baseplate cover	Inscopix	1050-002193
ProView™ Lens Probe; 0.6mm diameter, ~7.3mm length	Inscopix	100-000586
I/O box with XCAB-45BN cable	Noldus	N/A
Software and Algorithms		
ImageJ	https://imagej.nih.gov/ij/	RRID:SCR_003070
Prism 6.0	GraphPad Software	N/A
Adobe Illustrator CS6	Adobe	N/A
dfRTPA	In house written Python script	N/A
MC_Stimulus II	Multichannel systems	N/A
Vortran PC	Vortran Laser Technology	N/A
nVista	Inscopix	N/A
Mosaic	Inscopix	N/A
Ethovision XT with external control module	Noldus	N/A

Genotyping

PCR amplification of extracted genomic DNA was used to determine the genotype of the animals. 1 µl of genomic DNA was used as the template together with 0.25 µM primers in a commercial PCR reagent mixture (Reddymix PCR Master Mix, ThermoScientific). The presence of the SF1-Cre gene was detected by the resultant 250 bp PCR product using previously described pair of primers (Dhillon et al., 2006): forward 5' – CTGAGCTGCAGCGCAGGGACAT – 3' and reverse 5' – TGCGAACCTCATCACTCGTTGCAT – 3'. The reactions were carried out in a 10 µl final volume in a thermocycler (C100, BioRad) under the following cycling conditions: 95 °C for 3 min, followed by 5 cycles of 95 °C for 1 min, 58 °C for 1 min, and 72 °C for 2 min, then a further 25 cycles of 95 °C for 1 min, 56 °C for 1 min and 72 °C for 1 min, and finally 72 °C for 5 min. *Z/eg*-positive genotype was confirmed with a forward 5' –AAGTTCATCTGCACCACCG – 3' and reverse 5' – TCCTTGAAGAAGATGGTGCG – 3' primer pair producing a 750 bp product, with the same cycling conditions used as for the SF1-Cre genotyping. The PCR products were separated by electrophoresis in a 3% agarose gel prepared with TAE buffer (40 mM Tris acetate pH 8.3, 1 mM EDTA) and 0.02% ethidium bromide, and were subsequently imaged by ultraviolet light illumination.

Assessment of competing appetitive and avoidance-inducing drives

Customised assays were used to evaluate the influence of SF1 neurons on the conflicts between appetitive and avoidance motivations by providing a choice between safe but food-lacking or potentially dangerous and anxiogenic but food-containing environments. *Ad lib* fed animals were tested for 3 hours in open field and 4-chamber assays, where open, illuminated space and innate fear-inducing olfactory (2,4,5-trimethylthiazole or TMT, Sigma Aldrich) cues were used to create avoidance-inducing environments respectively (Genné-Bacon et al., 2016). Overnight fasted animals were evaluated in a fear-conditioning box (Med Associates) that involved mild (0.4 mA) electric shocks for the simulation of a dangerous environment.

Real-time place avoidance assay

The real-time place avoidance (RTPA) assay was performed in 2-chamber arena as described previously (Kunwar et al., 2015). After an initial 10 min exploration, blue light (488 nm, 20 Hz or 2 Hz tonic pulses of 1 ms) was delivered for 20 min when the mouse entered the pseudo-randomly designated stimulation chamber, followed by an additional 10 min of free-exploration. To quantify effects of varying stimulation frequencies, a dynamic frequency RTPA (dfRTPA) assay was used. After an initial 5 min, an in-house Python script initiated a 2 Hz photostimulation whenever the animals was in the designated chamber and increased stimulation frequency by 0.5 Hz every 5 min to a maximum of 6 Hz. Fed or overnight fasted SF1-ChR2 animals were tested in a crossover manner and preference of the animals. This task was further adjusted to evaluate pre-existing place preference by testing mice in a successive alleys (SA) arena, where the designated stimulation chamber was the least anxiogenic alley 1, or to analyse the competition between food seeking and SF1 stimulation-induced avoidance under different adiposity states. In the latter assay, empty pots were placed at the opposite ends of the 2-chamber RTPA arena, 1 in each of the chambers. Animals were calorie restricted (CR) to 85% of their initial bodyweight or fed either chow or high fat diet for 2 weeks. All groups were overnight fasted and after initial 5 min, food was placed in the pot of the stimulation chamber. After a further 5 min, a 2 Hz optogenetic stimulation protocol was initiated and proceeded to increase by 0.5 Hz every 5 min to 6 Hz as described for dfRTPA above.

Food intake

For fast-refeed studies, animals were fasted overnight from 17:00. For DREADD studies, animals were administered CNO 30 min before returning food (0.5 mg/kg for SF1-hM3Dq and 2.5 mg/kg for SF1-hM4Di animals). For optogenetic studies, mice were pre-stimulated for 5 min with continuous 1 ms, 2 Hz 488 nm laser light pulses and stimulation continued for 5 h. For food intake measurements in a fed state, animals were injected with CNO at 12:30. For studies in the BioDAQ system (Research Diets, Inc) CNO was delivered via IP injections at 12:30 and 18:30 and food intake, feeding bouts and time spent engaged with the food were recorded. Data was exported in 1 hour bins and analysed in Excel.

Body composition

For chronic CNO studies, body composition was determined using an EchoMRI™-500 analyser on the day that the treatment was started and then weekly for the duration of the studies. Measurements of fat

mass, lean mass, total and free water were acquired consistently between 8:00 and 14:00 with triple acquisition settings.

Assessment of locomotion

A circular open field (COF) arena, 40 cm diameter and 40 cm height, was used to assess spontaneous mobility. Movement velocity over a 10 min period was quantified 30 min and 5 h post-CNO-injection. To evaluate horizontal (X) and vertical (Z) locomotion in familiar environments, animals were housed in Comprehensive Lab Animal Monitoring System (CLAMS, Oxymax) for at least 3 days to ensure habituation. Just before the dark period at 19:00, CNO was administered into the drinking water and infrared beam brakes were quantified.

Assessment of anxiety

The elevated plus maze (EPM), successive alleys (SA), elevated zero maze (EZM) and light/dark box (LD) were used as a measure of anxiety-like exploratory behaviour, 30 min and/or 5 h after CNO administration (Bourin and Hascoët, 2003; Braun et al., 2011; Deacon, 2013; Pellow et al., 1985). Increased time spent in open and/or more brightly illuminated areas in these tests was considered as a mark of decreased anxiety-like behaviour.

Conditioned place preference

Conditioned place preference (CPP) was assessed in three chamber automated conditioned place preference boxes (Med Associates). After assessing pre-existing biases for one of the chambers (day 1), animals were randomly assigned to the chamber in which they would receive the CNO injection. Over the next 4 conditioning days, animals were injected with vehicle prior to confinement to one chamber in the morning and then injected with CNO and confined to the alternate chamber in the afternoon. On the 6th day, animals were placed in the centre chamber and allowed to freely explore the CPP box for 15 min. Time spent in the vehicle- and CNO-paired chambers was quantified.

Dissections for tissue collection

In order to collect tissues for the assays described below, mice were culled by cervical dislocation. Brains were collected and kept in 4% paraformaldehyde (PFA) overnight, before being transferred to 30% sucrose until no longer buoyant. Then brains were frozen on dry ice and maintained at -80 °C. Other tissues were frozen in liquid nitrogen immediately after harvesting and also kept at -80 °C.

Perfusion

Animals were overdosed with sodium pentobarbital (Euthatal) and intracardially perfused with 10-20 ml of sterile PBS'A' buffer, pH 7.2 and buffered 4% PFA. Brains were dissected and incubated overnight in 4% PFA, then transferred into 30% sucrose until no longer buoyant. The brains were then frozen in dry ice and kept at -80 °C.

Histology

For c-fos immunohistochemistry assessment of hM3Dq functionality, SF1-hM3Dq animals were injected with CNO or vehicle via the intraperitoneal (IP) route and perfused 90 min later. For functional assessment of optogenetic activation, SF1-ChR2 or SF1-eYFP animals were stimulated at 2 or 10 Hz for 90 min and perfused afterwards. For immunoreactivity detection, rabbit anti c-fos monoclonal antibodies (catalogue number: 2250 from Cell Signaling Technologies) were used in combination with chicken anti-rabbit IgG ((H+L), conjugated with Alexa Fluor 647, Molecular Probes, catalogue number: A-21443) secondary antibody. Firstly, coronal brain sections (35-100 µm thickness) were placed in meshed wells and washed in a bath of sterile PBS'A' buffer, pH 7.2, under gentle rocking. Then the sections were transferred to a blocking solution (1% chicken serum, 1 mg/ml bovine serum albumin (BSA) and 12.5% Triton-X) and incubated at room temperature (RT) for 1 h. Afterwards, sections were incubated in a 1:400 primary antibody solution, prepared in blocking mixture, for 1 h at RT and then overnight at 4 °C. The following day, sections were washed for 30 min and incubated in a 1:400 dilution of secondary antibody in the blocking solution for 2 h at RT. Then, sections were washed again for 30 min and mounted on slides using Shandon Immu-Mount (#9990402, Thermo Scientific) and imaged (TCS SP5, Leica). For detection of mCherry and/or eGFP brains were sectioned, mounted and imaged as described above.

References

Armbruster, B.N., Li, X., Pausch, M.H., Herlitze, S., and Roth, B.L. (2007). Evolving the lock to fit the key to create a family of G protein-coupled receptors potently activated by an inert ligand. Proc. Natl. Acad. Sci. *104*, 5163–5168.

Bourin, M., and Hascoët, M. (2003). The mouse light/dark box test. Eur. J. Pharmacol. *463*, 55–65.

Braun, A.A., Skelton, M.R., Vorhees, C. V, and Williams, M.T. (2011). Comparison of the elevated plus and elevated zero mazes in treated and untreated male Sprague-Dawley rats: effects of anxiolytic and anxiogenic agents. Pharmacol. Biochem. Behav. *97*, 406–415.

Deacon, R.M.J. (2013). The successive alleys test of anxiety in mice and rats. J. Vis. Exp.

Dhillon, H., Zigman, J.M., Ye, C., Lee, C.E., McGovern, R. a, Tang, V., Kenny, C.D., Christiansen, L.M., White, R.D., Edelstein, E. a, et al. (2006). Leptin directly activates SF1 neurons in the VMH, and this action by leptin is required for normal body-weight homeostasis. Neuron *49*, 191–203.

Genné-Bacon, E.A., Trinko, J.R., and DiLeone, R.J. (2016). Innate Fear-Induced Weight Regulation in the C57BL/6J Mouse. Front. Behav. Neurosci. *10*, 132.

Kunwar, P.S., Zelikowsky, M., Remedios, R., Cai, H., Yilmaz, M., Meister, M., and Anderson, D.J. (2015). Ventromedial hypothalamic neurons control a defensive emotion state. Elife *4*, 1–30.

Novak, A., Guo, C., Yang, W., Nagy, A., and Lobe, C.G. (2000). Z/EG, a double reporter mouse line that expresses enhanced green fluorescent protein upon Cre-mediated excision. Genesis *28*, 147–155.

Pellow, S., Chopin, P., File, S.E., and Briley, M. (1985). Validation of open : closed arm entries in an elevated plus-maze as a measure of anxiety in the rat. J. Neurosci. Methods *14*, 149–167.
