# Supplementary material for: Modulation of SF1 Neuron Activity Coordinately Regulates Both Feeding Behavior and Associated Emotional States
Source: Cell Rep. 2017 Dec 19;21(12):3559–72. doi: 10.1016/j.celrep.2017.11.089 (PMC5746599; doi:10.1016/j.celrep.2017.11.089)
Supplement: Document S2. Article plus Supplemental Information [file mmc2.pdf]

Cell Reports

Article

Modulation of SF1 Neuron Activity Coordinately Regulates Both Feeding Behavior and Associated Emotional States

Graphical Abstract

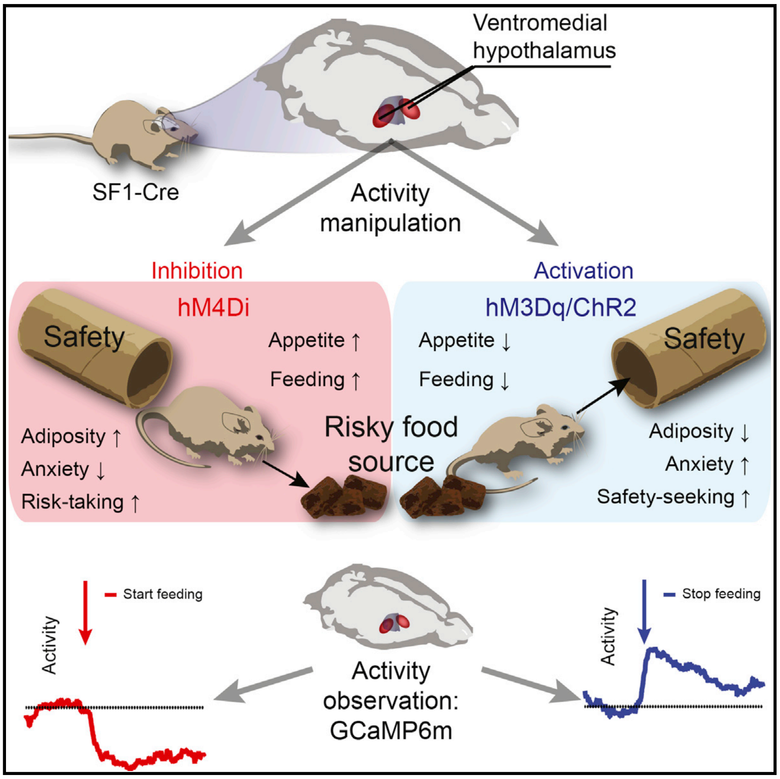

Highlights

- Intrinsic SF1 neuron activity is low during feeding and increases when feeding stops
- Manipulating SF1 neuron activity controls food intake and related behaviors
- Response to SF1 neuron activity is sensitive to energy status
- SF1 neural circuitry integrates feeding and emotional states

Authors

Paulius Viskaitis, Elaine E. Irvine, Mark A. Smith, ..., Mark A. Ungless, Graeme Milligan, Dominic J. Withers

Correspondence

d.withers@imperial.ac.uk

In Brief

Viskaitis et al. show that hypothalamic SF1 neurons act as a nutrient-sensitive switch between feeding and anxiety states. They identify a key circuit that permits feeding and related behaviors when its activity is low but primes the animal to stop feeding and to face potential stresses when activity is high.

Viskaitis et al., 2017, Cell Reports *21*, 3559–3572
December 19, 2017 © 2017 The Authors.
https://doi.org/10.1016/j.celrep.2017.11.089

CellPress

OPEN ACCESS
CellPress

Modulation of SF1 Neuron Activity Coordinately Regulates Both Feeding Behavior and Associated Emotional States

Paulius Viskaitis,[1,2] Elaine E. Irvine,[1,2] Mark A. Smith,[1,2] Agharul I. Choudhury,[1,2] Elisa Alvarez-Curto,[3] Justyna A. Glegola,[1,2] Darran G. Hardy,[1,2] Silvia M.A. Pedroni,[1,2] Maria R. Paiva Pessoa,[1,2] Anushka B.P. Fernando,[1,2] Loukia Katsouri,[1,2] Alessandro Sardini,[1,2] Mark A. Ungless,[1,2] Graeme Milligan,[3] and Dominic J. Withers[1,2,4,*]
[1]MRC London Institute of Medical Sciences, Du Cane Road, London W12 0NN, UK
[2]Institute of Clinical Sciences, Faculty of Medicine, Imperial College London, Du Cane Road, London W12 0NN, UK
[3]Centre for Translational Pharmacology, Institute of Molecular, Cell and Systems Biology, College of Medical, Veterinary and Life Sciences, University of Glasgow, Glasgow G12 8QQ, UK
[4]Lead Contact
*Correspondence: d.withers@imperial.ac.uk
https://doi.org/10.1016/j.celrep.2017.11.089

SUMMARY

Feeding requires the integration of homeostatic drives with emotional states relevant to food procurement in potentially hostile environments. The ventromedial hypothalamus (VMH) regulates feeding and anxiety, but how these are controlled in a concerted manner remains unclear. Using pharmacogenetic, optogenetic, and calcium imaging approaches with a battery of behavioral assays, we demonstrate that VMH steroidogenic factor 1 (SF1) neurons constitute a nutritionally sensitive switch, modulating the competing motivations of feeding and avoidance of potentially dangerous environments. Acute alteration of SF1 neuronal activity alters food intake via changes in appetite and feeding-related behaviors, including locomotion, exploration, anxiety, and valence. In turn, intrinsic SF1 neuron activity is low during feeding and increases with both feeding termination and stress. Our findings identify SF1 neurons as a key part of the neurocircuitry that controls both feeding and related affective states, giving potential insights into the relationship between disordered eating and stress-associated psychological disorders in humans.

INTRODUCTION

Feeding involves a complex series of actions that an animal must undertake in order to procure nutrients in a timely and safe manner. In the wild, feeding is often associated with exploration of novel and potentially dangerous environments. As a result, the systems controlling feeding behavior have evolved under a requirement for swift selection of situationally appropriate behaviors and accurate metabolic tuning. This involves integration of internal homeostatic signals, external environmental stressors, motivational drives, and learned associations to consolidate conflicts between risk mitigation and the requirement to feed. Consistent with these observations, the neurocircuitry and whole-body physiological processes underlying energy homeostasis have substantial functional overlap with the systems controlling responses to stressful situations (Sweeney and Yang, 2017; Ulrich-Lai and Ryan, 2014). For example, arcuate nucleus agouti-related peptide (AgRP), lateral hypothalamic, and amygdalar neurons have important roles in the incorporation of various emotional aspects into the regulation of feeding (Sweeney and Yang, 2017). In experimental models and humans, both disordered eating behavior and obesity have a significant association with stress-related behavioral changes, such as anxiety and depression (Anderson et al., 2001; Faith et al., 2002; Gariepy et al., 2010; Hryhorczuk et al., 2013; Stunkard et al., 2003).

The ventromedial hypothalamus (VMH) is a key brain region involved in the control of feeding, metabolism, and the manifestation of stress and anxiety-related behaviors. Lesioning and electrical stimulation studies implicate the VMH in the regulation of feeding, body weight, and defensive behaviors (King, 2006), and a role in the link between appetitive and affective responses has also been suggested (Grossman, 1966). Recent studies manipulating nutrient- and hormone-sensing-signaling pathways in steroidogenic factor 1 (SF1) neurons have confirmed the role of the VMH in the regulation of body weight and metabolism. However, somewhat surprisingly, the studies have in general not detected effects on acute food intake (Cardinal et al., 2014; Cheung et al., 2015; Dhillon et al., 2006; Kim et al., 2012; Klöckener et al., 2011; Ramadori et al., 2011; Xu et al., 2010, 2011). Pharmacogenetic and optogenetic manipulations of SF1 neurons have shown that these cells regulate defensive social states (Silva et al., 2013, 2016) and profound escape behaviors (Kunwar et al., 2015; Wang et al., 2015), and a recent study has shown that the activation of SF1 neurons can suppress acute feeding but through indeterminate mechanisms (Coutinho et al., 2017). Despite these observations, the precise relationships among SF1 neuronal activity, acute feeding regulation, and other behaviors associated with the VMH function remain unclear.

Here we find that SF1 neurons alter feeding via direct changes in appetite and by altering locomotion, exploration, anxiety,

Check for updates

This is an open access article under the CC BY license (http://creativecommons.org/licenses/by/4.0/).

valence, memory, and risk-taking. We demonstrate an SF1 neuron firing frequency-dependent switch between feeding and anxiety-related behavior that is also sensitive to energy stores, with low intrinsic SF1 activity being permissive for feeding while increased activity occludes food intake. Thus, we identify a key integrative role for SF1 neurons in the regulation of feeding and associated behavioral states.

RESULTS

Firing Pattern Characteristics and Behavioral Outcomes of Optogenetic Activation of SF1 Neurons Depend on Stimulation Frequency

To activate VMH SF1 neurons, we injected adeno-associated virus (AAV) particles containing a Cre-dependent, mCherry-fused channelrhodopsin (ChR2) virus (AAV1-DIO-ChR2-mCherry) into the VMH of SF1-Cre mice to produce SF1-ChR2 animals. We used Cre-negative littermates and/or AAV1-DIO-EYFP-injected SF1-Cre mice as controls. mCherry or EYFP was expressed in the VMH, with more than 80% of SF1 neurons expressing ChR2 (Figure S1A; data not shown). In acute VMH slice patch recordings, tonic 1-ms, 2-Hz photo-stimulation produced spiking with 100% fidelity, preserved some endogenous action potentials, and modestly increased firing frequency (Figure S1B; data not shown). *In vivo*, this firing frequency was associated with increased c-*fos* expression in the VMH, indicating neuronal activation (Figures S1C and S1D). However, with photo-stimulation frequencies of 5 Hz or higher in slice preparations, the endogenous action potential firing pattern was occluded and replaced by an optogenetically driven firing pattern (Figure S1B). The c-*fos* expression was significantly higher with *in vivo* 10-Hz stimulation than in control or 2-Hz-stimulated SF1-ChR2 animals (Figures S1C and S1D). Thus, higher-frequency optogenetic stimulation (>5 Hz) overrode intrinsic firing patterns, and it resulted in significantly increased neuronal recruitment *in vivo*.

High-frequency optogenetic stimulation (>20 Hz) of SF1 neurons causes profound defensive responses, including freezing and escape attempts. We observed these behaviors at similar stimulation frequencies (Figure S1E; data not shown). However, steady-state SF1 firing frequency in *ex vivo* slice electrophysiology studies ranges between 3.5 and 6.2 Hz (Kim et al., 2008; Klöckener et al., 2011), consistent with our observations (6.2 ± 0.9 Hz; Figure S1F). Nevertheless, brief high-frequency bursts of spiking could be observed >20 Hz. This action potential firing pattern was also seen during *in vivo* recordings of VMH neurons, which had a steady-state firing frequency of 3.5 ± 0.9 (Figure S1F). Although high-frequency bursts of SF1 neuronal activity may underlie a particular behavioral response (e.g., escape from imminent predator threat), we reasoned that the frequencies typically used to date in optogenetic analyses of SF1 neuron control of behavior may not reflect the physiological role of this neuronal population during non-threatening conditions. Therefore, we examined the behavioral consequences of optogenetically altering SF1 neuronal activity across a range of firing frequencies seen under steady-state conditions. In a real-time place avoidance (RTPA) assay, we confirmed that high-frequency (20-Hz) optogenetic stimulation of SF1 neurons was aversive, with mice avoiding the stimulation chamber (Figures 1A and 1B). In contrast, low-frequency stimulation of 2 Hz was not aversive, as mice spent equal amounts of time in each chamber (Figure 1C).

To address the behavioral outcomes of different stimulation frequencies, we used a 2-chamber dynamic frequency RTPA (dfRTPA) assay, exposing mice to varying optogenetic frequencies in the designated stimulation chamber. The 2-Hz optogenetic stimulation started 5 min after the initial exploration, and stimulation frequency was increased by 0.5 Hz every 5 min to a maximum of 6 Hz. At low frequencies, mice showed no preference for either chamber, but, at frequencies of 4 Hz or higher, a significant aversion to the stimulated chamber was observed, and avoidance increased linearly with stimulation frequency (Figure 1D).

To determine if optogenetic stimulation-induced avoidance overcomes a pre-existing place preference, we used the dfRTPA protocol in a successive alley (SA) apparatus. Mice were only exposed to the optogenetic stimulation when they were in the preferred safe zone (alley 1). Low-frequency stimulation did not affect place preference, but increasing stimulation frequency resulted in a linear increase in avoidance of the preferred enclosed dark area of alley 1 (Figure 1E). Therefore, we observed a stimulation frequency threshold in SF1 neurons, where frequencies below 4 Hz were not significantly aversive, while higher frequencies induced active avoidance overcoming pre-existing place preference.

In the RTPA studies, mice terminated optogenetic stimulation by leaving the stimulation chamber. To test whether a more sustained low-frequency stimulation caused changes in locomotion, we used a square open field arena and exposed mice to either 2- or 5-Hz stimulation for 1 hr. Prolonged stimulation at 5 Hz, but not at 2 Hz, significantly increased locomotion (Figure 1F).

Low-Frequency Optogenetic Stimulation of SF1 Neurons Suppresses Feeding in the Absence of Detectable Anxiety-Associated Behavior

The absence of behavioral changes with 2-Hz frequency stimulation of SF1 neurons prompted us to examine feeding at this frequency. The 2-Hz stimulation over a 5-hr period significantly suppressed feeding after a fast (Figure 1G), while virus expression or light administration alone had no effect. Cumulative 24-hr food consumption was not different between the groups, indicating reversibility of the suppressive effects upon feeding (Figure S1G). During an identical stimulation protocol, mice filmed in a home cage environment (Figure 1H) showed unaltered velocity and time spent in the shelter area, but they spent significantly less time in the feeding zone, suggesting reduced appetitive behavior (Figures 1I–1K). To exclude low-level stress as measured by autonomic activation as a cause of suppressed feeding, we measured brown adipose tissue temperature and heart rate (HR). While these were unaltered with 2-Hz optogenetic stimulation, 5-Hz stimulation increased temperature but lowered HR (Figures S1H and S1I). In summary, 2-Hz optogenetic stimulation of SF1 neurons activated this population and suppressed feeding, without affecting locomotion, avoidance, or autonomic activity. In contrast, optogenetic firing frequencies of 4 Hz and above resulted in increased avoidance-related and defensive behaviors.

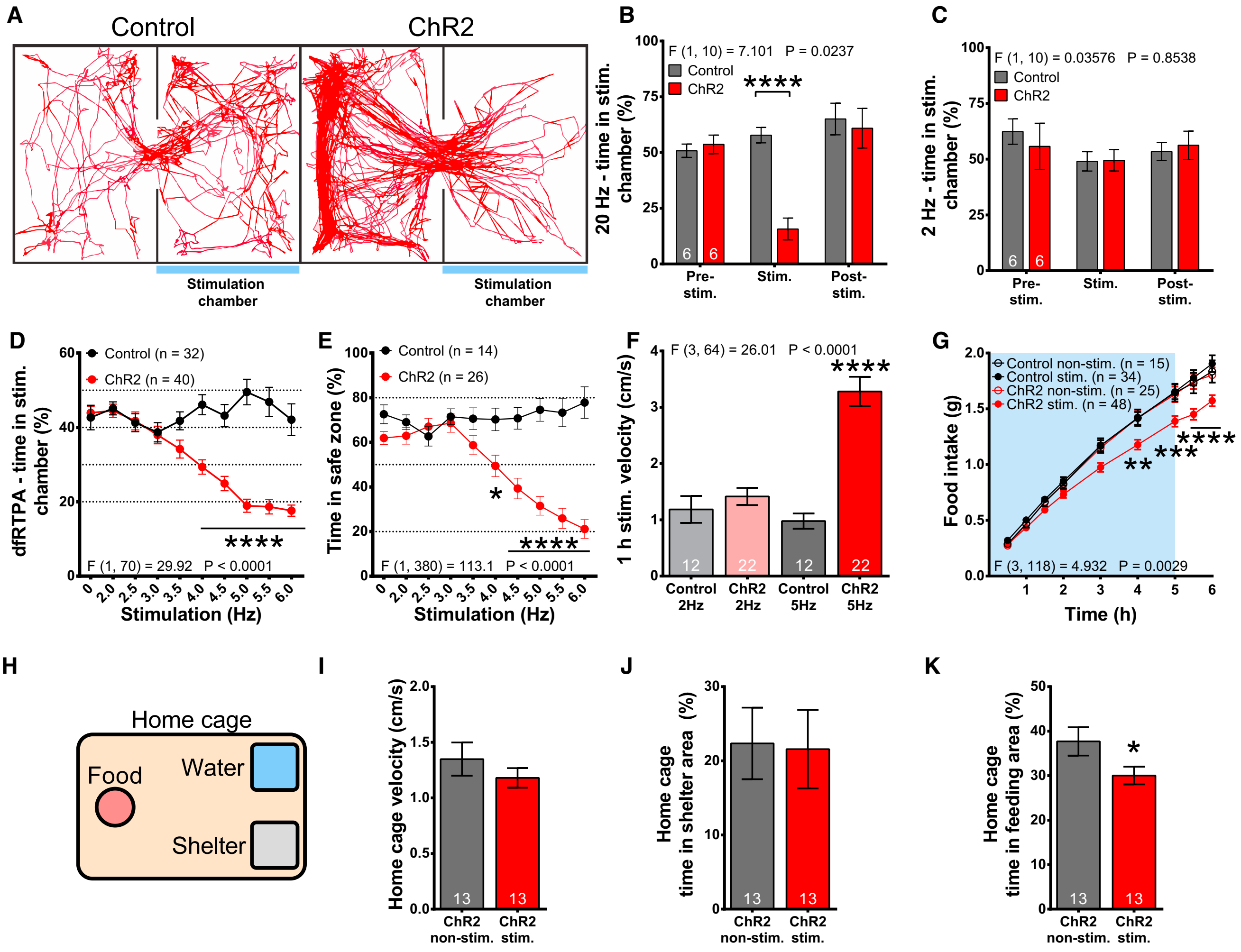

Figure 1. SF1 Neurons Display Optogenetic Stimulation Frequency-Dependent Divergence of Defensive and Feeding Behaviors

(A) Representative movement traces in 20-Hz real-time place aversion (RTPA) assay.

(B) Time spent in stimulation chamber in 20-Hz RTPA assay.

(C) Same as in (B), but with 2-Hz stimulation.

(D) Time spent in the stimulation chamber during the dynamic frequency RTPA assay (dfRTPA).

(E) Similar to (D), but conducted in successive alley (SA) arena, with alley 1 as the designated stimulation chamber.

(F) Locomotion in square open field during 1 hr of continuous 2- or 5-Hz optogenetic stimulation.

(G) Fasted food intake during 2-Hz optogenetic stimulation.

(H) Depiction of home cage used in (I)–(K).

(I) Locomotion during 5-hr-long, 2-Hz optogenetic stimulation.

(J) Time spent in shelter area.

(K) Time spent in feeding area.

Data are expressed as mean values ± SEM. 2-way ANOVA, repeated-measures (RM), followed by Sidak post hoc tests were performed on (B)–(G), and for (I)–(K) a paired t test was used (*p < 0.05, **p < 0.01, ***p < 0.001, and ****p < 0.0001). See also Figure S1.

Pharmacogenetic Modulation Results in Changes in the Activity of SF1 Neurons

To explore the effects of altering SF1 neuron activity under potentially more physiological firing patterns, we used hM3Dq and hM4Di designer receptors exclusively activated by designer drugs (DREADDs) to excite or inhibit SF1 neurons, respectively. Transduction efficiencies of greater than 80% were observed with both viruses (Figure S2A; data not shown). *Ex vivo* electrophysiology showed that SF1-hM3Dq neurons were reversibly depolarized upon brief application of clozapine-N-oxide (CNO) (Figures 2A and 2B), while SF1-hM4Di neurons were reversibly hyperpolarized and silenced (Figures 2A and 2C). *In vivo* VMH neuron spiking frequency was increased in SF1-hM3Dq mice (Figures 2D, 2F–2H, and S2B) and reduced in SF1-hM4Di animals after CNO injection (Figures 2E–2H). While pharmacogenetically induced changes in the steady-state firing frequency were modest (<2 Hz) compared to optogenetic stimulation, intrinsic firing patterns were preserved

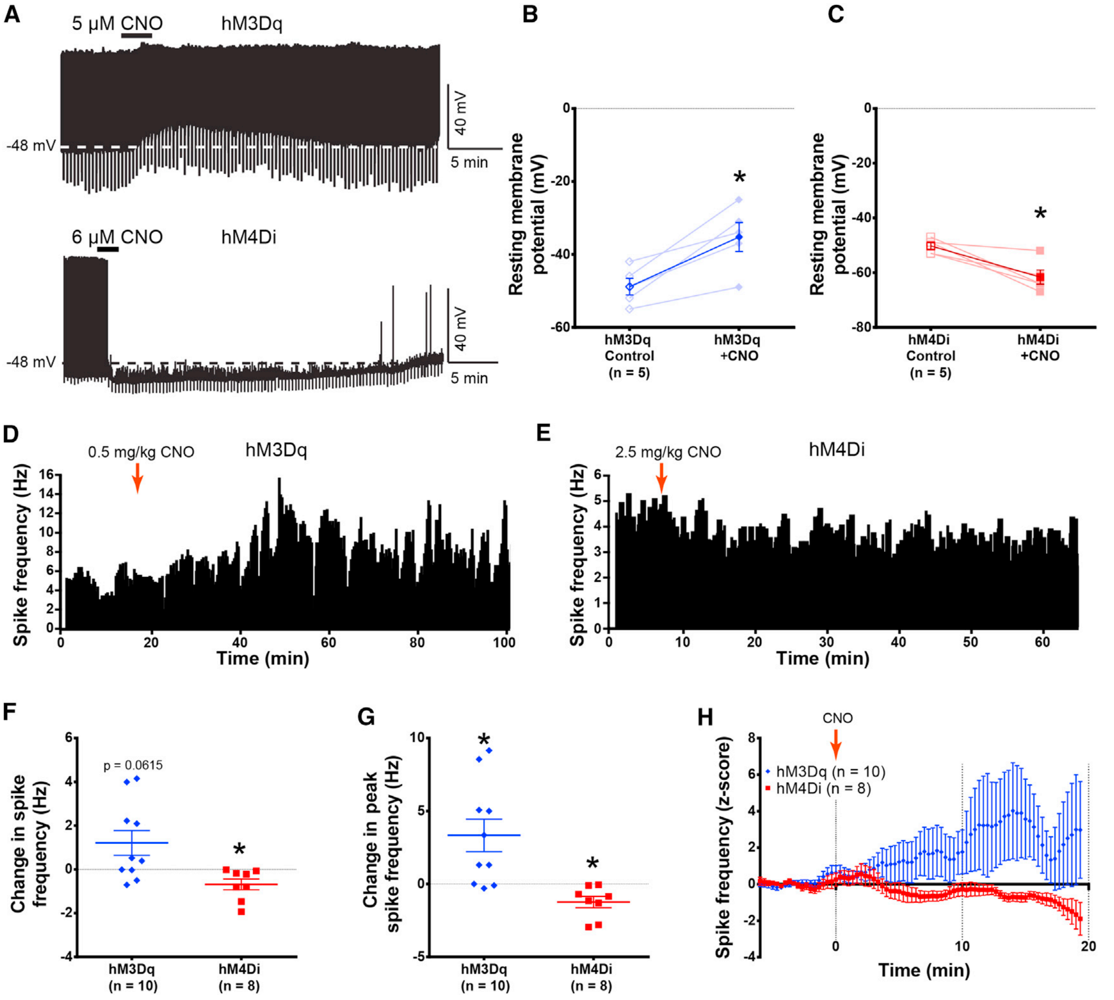

Figure 2. Effects of hM3Dq and hM4Di on SF1 Neuron Electrophysiology

(A) Representative trace of slice electrophysiology in SF1-hM3Dq and SF1-hM4Di neurons.

(B and C) CNO induced changes in resting membrane potential in SF1-hM3Dq (B) and SF1-hM4Di (C) neurons *ex vivo* (n = 5).

(D and E) Representative, 20-s-bin histograms of a VMH neuron firing frequency in SF1-hM3Dq (D) and SF1-hM4Di (E) mice during *in vivo* electrophysiology recordings.

(F) Changes in average firing frequency during *in vivo* recordings.

(G) Changes in peak firing frequency during the experiment in (F).

(H) *Z* score representation of the changes to the firing frequency during the experiment in (F).

Data are expressed as mean values ± SEM. A paired t test was used for (B) and (C), and 1-sample t tests were used for (F) and (G) (*p < 0.05). See also Figure S2.

and CNO administration increased the expression of c-*fos* in SF1-hM3Dq animals, indicating *in vivo* activation (Figures S2C and S2D).

Acute DREADD-Mediated Activation or Inhibition of SF1 Neuronal Activity Regulates Feeding, and Chronic Modulation Alters Fat Mass

The activation of SF1 neurons in fasted SF1-hM3Dq mice suppressed feeding in both female and male mice (Figures 3A and 3B). In contrast, inhibition of SF1 neurons in SF1-hM4Di mice increased cumulative food intake in *ad libitum*-fed mice of both sexes (Figures 3C and 3D). Control virus expression or CNO did not alter feeding (data not shown). Under freely feeding conditions, SF1-hM3Dq mice ate significantly less, while SF1-hM4Di mice consumed more food compared to controls (Figure 3E), and this was associated with consistent changes in the number of feeding bouts and the time spent feeding (Figures S3A and S3B). In SF1-hM3Dq mice, we observed none of the dramatic acute escape or defensive behaviors seen with high-frequency optogenetic activation of SF1 neurons.

Next, we administered CNO continuously for 3 weeks in the drinking water (50 mg/L), and we measured body composition weekly. Whole-body fat was unaltered in the control animals of either gender, but in both sexes SF1-hM3Dq animals lost while SF1-hM4Di mice gained significant fat mass (Figures 3F and 3G). Taken together, these observations show that acute or

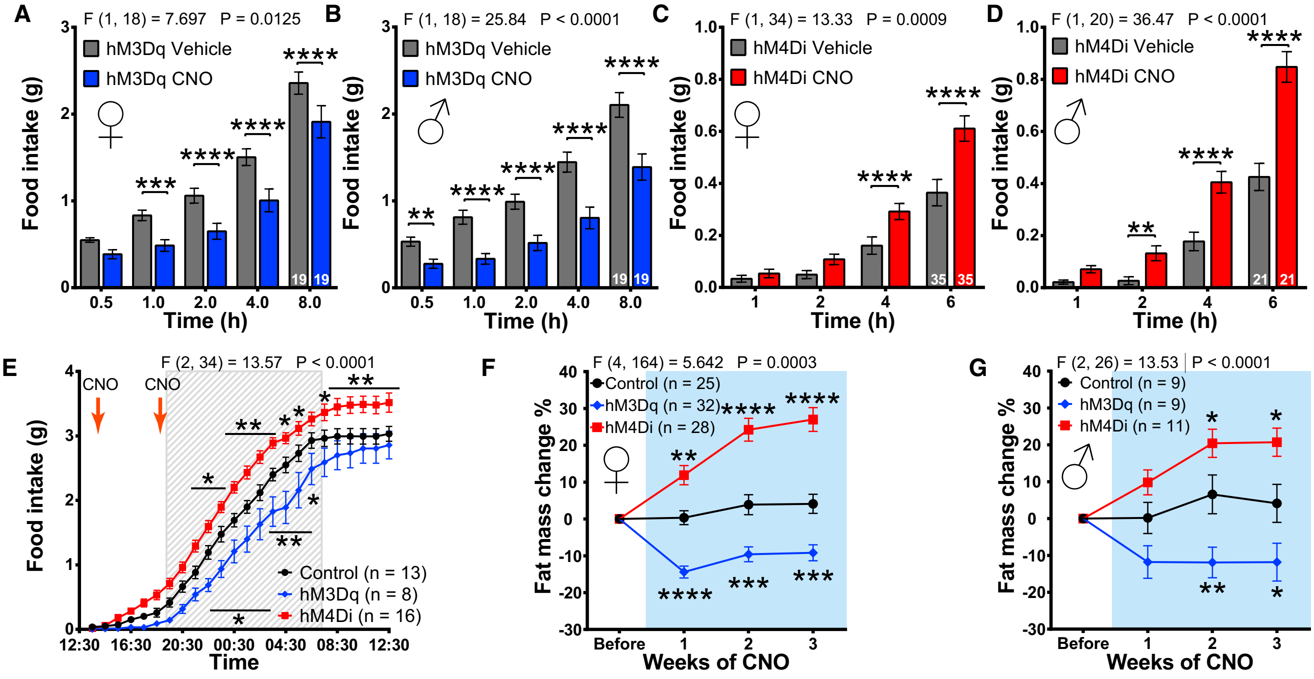

Figure 3. Pharmacogenetic Manipulation of SF1 Neuronal Activity Influences Acute Feeding and Sub-chronic Adiposity

(A and B) Fasted food intake after CNO administration in female (A) and male (B) SF1-hM3Dq mice.

(C and D) *Ad libitum* feeding in female (C) and male (D) SF1-hM4Di mice after CNO administration.

(E) Cumulative food intake in the BioDAQ automated feeding system.

(F and G) Changes in female (F) and male (G) whole-body adipose mass after 3 weeks of continuous CNO administration via the drinking water.

Data are expressed as mean values ± SEM. 2-way ANOVA, RM, followed by Sidak post hoc tests were used for analysis (*p < 0.05, **p < 0.01, ***p < 0.001, and ****p < 0.0001). See also Figure S3.

chronic modulation of SF1 neuronal activity has effects on feeding and adiposity.

SF1 Neurons Regulate Affective States that Impact on Feeding Behavior and Appetite

Next, we systematically probed the potential role of SF1 neurons to influence both the homeostatic and emotional/affective systems that regulate feeding (Figure 4A). In a circular open field (COF) task to assess locomotion as a measure of exploratory behavior, 30 min following CNO administration the SF1-hM3Dq mice had decreased activity, while SF1-hM4Di mice moved more than their respective controls (Figures 4B and 4C). These differences did not persist at 5 hr after CNO injection. We then assessed CNO effects on locomotion and exploration in the presence of food by measuring horizontal and vertical movement in a home cage environment. SF1-hM3Dq mice displayed reduced locomotion and rearing (Figures 4D and 4E), a measure of diversive exploration, while inhibition of SF1 neurons in SF1-hM4Di mice led to significant increases in both measures of exploratory behavior (Figures 4D and 4E). Modulation of SF1 neuronal activity, therefore, altered locomotion and exploration, behaviors relevant to food acquisition, in both novel and familiar environments.

Next, we determined the effects of altering SF1 activity upon behavior in novel anxiogenic environments. At 30 min after CNO administration, exploration of the anxiogenic areas by SF1-hM3Dq animals was significantly reduced in the elevated zero maze (EZM) assay (Figure 4F), but it was not statistically different from controls in the light/dark (LD; Figure 4H), elevated plus maze (EPM; Figure S4A), or SA (Figures S4B and S4C) tasks suggesting a mild anxiogenic phenotype. In contrast, the inhibition of SF1 neurons in SF1-hM4Di mice was highly anxiolytic, as these animals had significantly increased exploration of the anxiogenic areas of the EZM (Figure 4G), LD (Figure 4I), EPM (Figure S4A), and SA (Figure S4D). Furthermore, SF1-hM3Dq and SF1-hM4Di mice displayed alterations in locomotion with the same directionality and kinetics as seen for the changes in exploration of the open arm in the EZM assay (Figures S4E and S4F).

To assess if SF1 neuronal activity regulates valence and memory, animals were tested for conditioned place preference (CPP) after undergoing a 4-day conditioning protocol. The activation of SF1 neurons did not alter CPP (Figure S4G). In contrast, SF1-hM4Di mice displayed a significant preference for the CNO treatment chamber (Figure S4H). Thus, inhibition of SF1 neurons may produce positive valence and memory formation to the associated cues.

Next, we adapted a square open field arena to simultaneously assess the competing drives of appetite and avoidance. Testing took place in a dimly lit room, while the central region of the arena was illuminated by a bright overhead light (Figure 4J). Two pots were placed in the central illuminated area, one containing a pellet of chow and the other a similar sized non-edible object

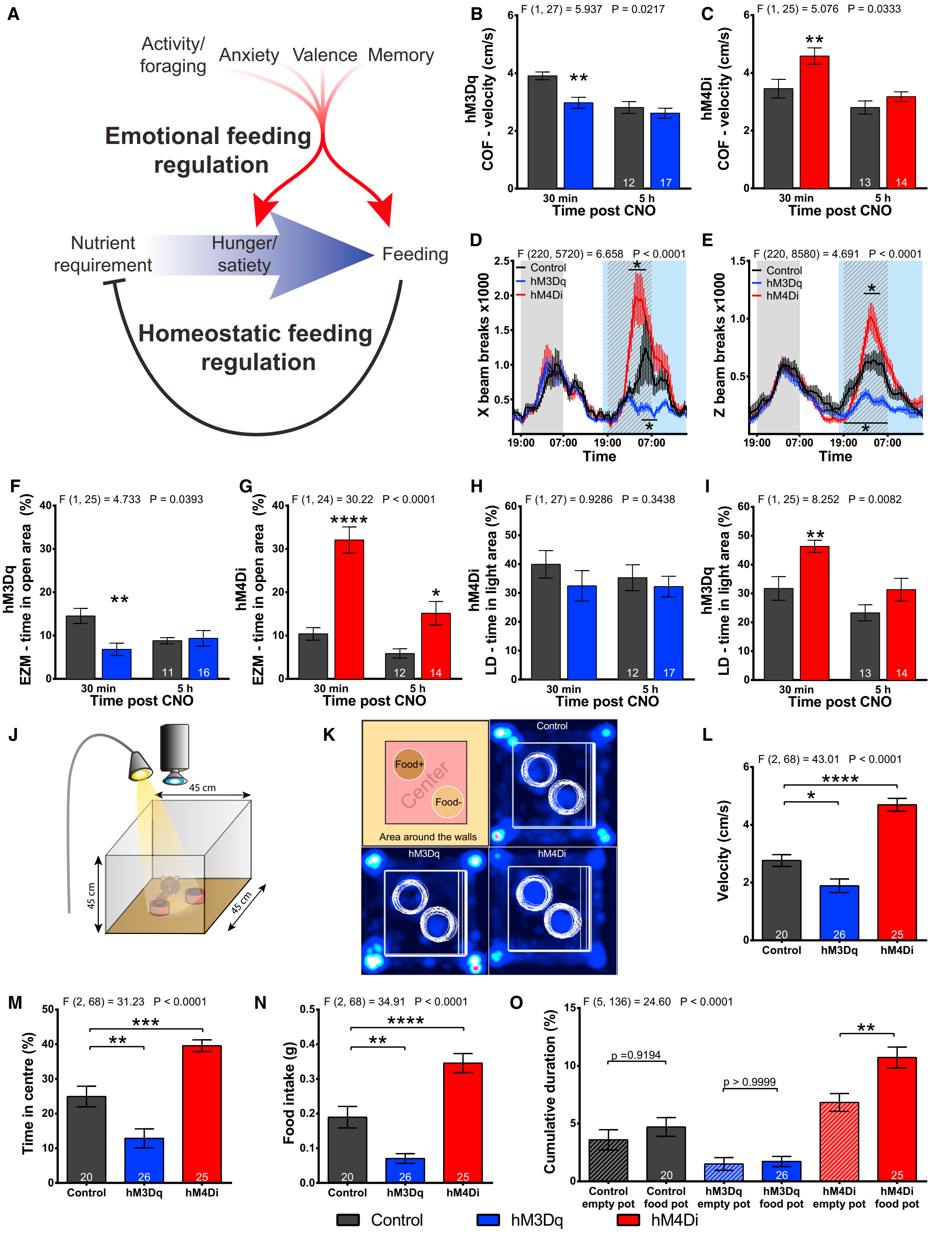

(legend on next page)

(Figure 4K). *Ad libitum*-fed mice were tested and received CNO 30 min before the task. SF1-hM3Dq mice had reduced locomotion (Figure 4L), exploration of the central area of the arena (Figure 4M), and food intake (Figure 4N), while SF1-hM4Di mice displayed the opposite effects. Furthermore, while control and hM3Dq mice spent equivalent amounts of time at the empty pot and the food pot, hM4Di mice spent increased time at the food pot, suggesting an increase in appetite (Figures 4K and 4O). Importantly, no gender-specific differences were detected for behaviors assessing affective states.

Inhibition of SF1 Neurons Facilitates the Extinction of Negative Conditioning during Feeding and Dampens Innate Odor-Induced Fear Avoidance of Food

Next, we determined the role of SF1 neuron activity on risk-taking behavior for food during either negative conditioning or exposure to an olfactory innate fear-inducing cue. In a task based on fear conditioning, naive, overnight-fasted animals were able to choose between three locations: an area with a food pot, a central insulated safety area, and an area with a non-edible object (Figure 5A). The test lasted for 40 min, with mild (0.4 mA) electric shocks delivered between 10:00 and 19:59 min. Mice could avoid the shocks by staying in the central area (Figure 5B). Throughout the test, control and SF1-hM4Di mice preferred the food area to the non-edible object area, but this was absent in the SF1-hM3Dq mice (Figure 5C). During the negative-conditioning phase, all three groups avoided the metal-grid area, spending more time in the safety area (Figure 5D). Upon discontinuation of the shocks, the inhibition of SF1 neurons facilitated extinction of negative conditioning, as the SF1-hM4Di mice spent less time in the safety zone compared to the control group and to the conditioning phase. Control animals showed a trend toward reduced time in the safety zone during the extinction period, but the SF1-hM3Dq mice did not display any signs of punishment extinction. Similar trends were observed when the food zone was considered (Figure S5A). Together these results suggest that SF1 neurons can regulate the extinction of short-term negative conditioning during feeding.

To examine whether SF1 neuron activity alters food seeking and feeding under olfactory avoidance-inducing conditions, we designed an assay using the conflicting motivation of the appetitive drive toward food and the avoidance of the innate fear-inducing cue, TMT (2,3,5-Trimethyl-3-trimethylthiazole; Figure 5E). The arena consisted of four chambers, each containing a pot. Two pots were scented with TMT (S+) and two were non-scented (S−), and, of these, one scented and one non-scented pot also contained food (F+; Figure S5B). Modulation of SF1 neurons had effects on the overall investigation of the pots (Figure S5B), with SF1-hM3Dq mice interacting less and SF1-hM4Di interacting more than the control animals (Figure 5F). Considering each of the pots separately, time spent at the non-scented, food containing pot (F+S–) was significantly increased in hM4Di and decreased in hM3Dq groups compared to controls (Figure 5F), while SF1-hM4Di mice also consumed significantly more food from this pot than the controls (Figure S5C). Importantly, SF1-hM4Di mice spent significantly more time at, and consumed more food from, the food-containing, TMT-scented pot (F+S+; Figures 5F and 5G) compared to controls, suggesting that the inhibition of SF1 neurons enabled SF1-hM4Di animals to partially overcome TMT avoidance when seeking food. However, the opposite effect in the SF1-hM3Dq mice was not significant (Figures 5F and 5G). No differences for the interaction with the TMT-smelling, food-lacking pot (F−S+) were detected for either of the experimental groups (Figure 5F), suggesting that SF1 neurons do not regulate avoidance of TMT in the absence of food. Lastly, exploration of the empty, unscented pot (F−S−) was not different between control and hM4Di groups, but it was reduced in SF1-hM3Dq mice (Figure 5F), demonstrating decreased motivation for exploration and interaction. Modulation of SF1 neurons did not affect the relative place preference between the different pots, except for the F−S− pot (Figure S5D). Therefore, even under potentially adverse environmental conditions that suppressed food intake, the inhibition of SF1 neurons led to significantly attenuated avoidance of the TMT-scented food pot and increased food consumption from this source. These findings suggest that SF1 neuron activity can shift the balance of the competition between food seeking and avoidance of innate fear cues but potentially via alterations in explorative behavior and appetite rather than the avoidance of TMT per se.

Nutrient Deprivation Blunts Avoidance Elicited by Optogenetic Activation of SF1 Neurons

SF1 neurons are sensitive to feeding state and metabolic signals, such as leptin, insulin, and glucose (Boden et al., 1989; Cardinal et al., 2014; Klöckener et al., 2011; Sternson et al., 2005). We explored whether nutrient status influenced escape or defensive behaviors, reasoning that animals in a state of negative energy

Figure 4. SF1 Neurons Regulate Appetite and Associated Affective States that Impact on Feeding

(A) Model of the interplay between homeostatic and affective pathways that influence feeding.

(B and C) Locomotion of SF1-hM3Dq (B) and SF1-hM4Di (C) mice in circular open field (COF) 30 min and 5 hr after CNO administration.

(D) Moving average (8 time points) of horizontal infrared beam breaks in a home cage environment.

(E) Similar to (D), but vertical beam breaks were assessed.

(F and G) Time spent by SF1-hM3Dq (F) and SF1-hM4Di (G) mice in the anxiogenic open areas in the elevated zero maze (EZM) task 30 min and 5 hr after CNO administration.

(H and I) Time spent in anxiogenic light area of the light/dark arena (LD) by SF1-hM3Dq (H) and SF1-hM4Di (I) mice.

(J) Depiction of the behavioral task used in (K)–(O).

(K) Area subdivision of the task in (J) and place preference heatmaps of CNO-injected, *ad libitum*-fed animals.

(L–O) Velocity (L), time spent in the illuminated center area (M), food intake (N), and time spent near food or non-edible object (O) during the experiment shown in (K).

1-way ANOVA with Sidak post hoc was used to analyze (L)–(N), and 2-way ANOVA, RM, Sidak tests were performed on the remaining panel (*$p < 0.05$, **$p < 0.01$, ***$p < 0.001$, and ****$p < 0.0001$). See also Figure S4.

OPEN ACCESS
CellPress

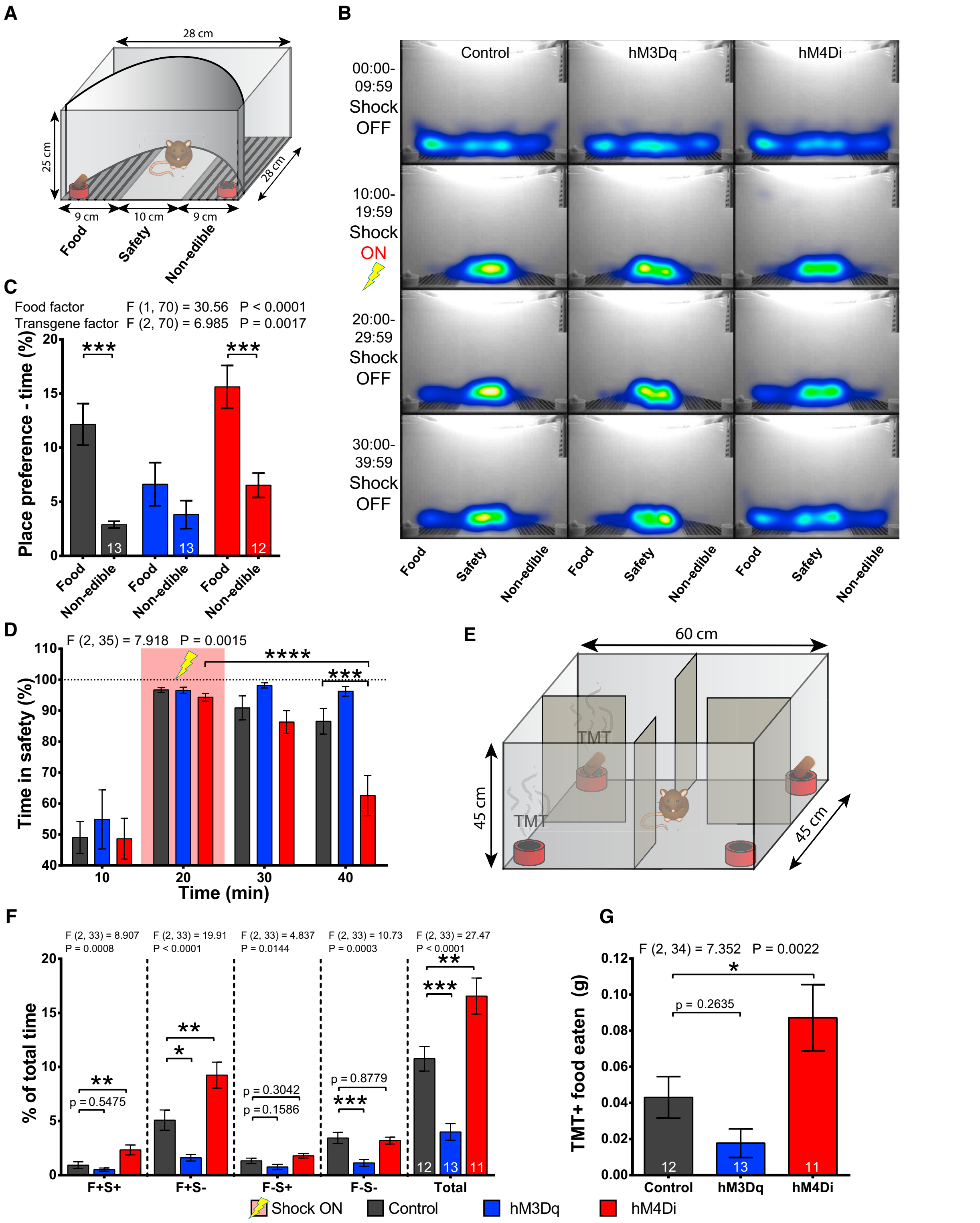

Figure 5. SF1 Neurons Regulate the Balance between Food Seeking and Avoidance of Negative Conditioning or TMT

(A) Depiction of the negative feeding-conditioning assay.

(B) Time spent heatmaps in 10-min bins.

(C) Time spent in the food and non-edible object areas during the experiment in (B).

(D) Time spent in the safe zone per 10-min bins from the experiment in (B).

(legend continued on next page)

balance might be more resistant to the anxiogenic effects of SF1 activation. In the two-chamber dfRTPA assay (Figures 1E and S6A), fasting significantly blunted the avoidance caused by optogenetic stimulation of SF1 neurons (Figure 6A). With food added to the stimulation chamber to provide a motivational goal to enter the chamber (Figure 6B), there were significant effects of adiposity upon the ability of SF1 activation to drive avoidance behavior (Figure 6C). In the fasted state, calorie-restricted mice (Figure S6B) were less sensitive, while high-fat diet (HFD)-fed animals were more sensitive (Figure S6D) to the stimulation-induced avoidance of the food chamber than the chow-fed mice (Figure S6C). These findings suggest that SF1 neurons, or the neurocircuitry engaged by them, are sensitive to nutritional state and, when the need for nutrients is increased, the ability of SF1 neurons to induce a feeding-suppressing affective behavioral state is blunted.

Food Detection Lowers and an Anxiogenic Stimulus Elevates SF1 Activity in Freely Moving Mice

The preceding experiments suggest that suppressing SF1 activity is permissive for feeding while increasing activity occludes food intake. To directly monitor SF1 neuronal activity during feeding-related and stress-induced behavior, we recorded fluorescence in SF1 neurons expressing a calcium reporter of neuronal activity (Figure 6D). Food presentation to fasted mice showed that SF1 activity decreased upon approach to food, while leaving food was associated with increased activity (Figures 6E and 6F). The activity changes were highly specific to food, as approaching or leaving an empty food pot or presentation of a non-edible object had little effect on SF1 activity (Figures S6E–S6G). Furthermore, the stressful experience of a foot shock elicited a marked activation of SF1 neurons that was significantly greater than feeding termination (Figures 6E and 6F). Therefore, consistent with our optogenetic and chemogenetic studies, feeding behavior was associated with low while anxiety was associated with high intrinsic SF1 activity.

DISCUSSION

Hypothalamic and limbic neuronal populations not only regulate feeding but also control related motivated behaviors and affective states (Sweeney and Yang, 2017; Ulrich-Lai and Ryan, 2014). VMH neurons also control food intake and other behavioral outputs, and, therefore, they may subserve a similar integrative role, but to date there has been no rigorous attempt to study such an interaction. Here, using systematic behavioral and physiological phenotyping platforms combined with methods to acutely alter or measure neuronal activity, we show that SF1 neurons (the predominant VMH population) alter food intake via changes in appetite and feeding-related behaviors, identifying a key part of the neurocircuitry controlling both ingestive behavior and related affective states.

SF1 Neurons Regulate Acute Food Intake in an Activity-Dependent Manner

We show that optogenetic stimulation of SF1 neurons, at typical steady-state frequencies, acutely inhibits food intake. Likewise, chemogenetic stimulation reduces while chemogenetic suppression of SF1 neurons increases food intake, demonstrating acute regulation of feeding behavior by this neuronal population. Consistent with these induced changes, SF1 activity measured in freely moving mice was lower when they engaged with food and was higher under stressful conditions.

While these findings align with historical studies utilizing electrical stimulation of VMH neurons to inhibit food intake, and with VMH-lesioning studies that were associated with marked hyperphagia, these approaches lack cellular precision and may have stimulated or damaged fibers that traverse the VMH (King, 2006). Therefore, some of the roles ascribed to the VMH may have been due to effects on cell populations outside the VMH. More recent genetic studies specifically targeting hormone- and nutrient-signaling pathways in VMH SF1 neurons have demonstrated key roles in body weight regulation, but somewhat surprisingly they have not revealed effects on feeding (Cardinal et al., 2014; Cheung et al., 2015; Dhillon et al., 2006; Kim et al., 2012; Klöckener et al., 2011; Ramadori et al., 2011; Xu et al., 2010, 2011). In contrast, our data directly establish an inverse relationship between SF1 neuron activity and acute food intake. That such effects are relevant to energy homeostasis is suggested by the changes in adiposity seen with sub-chronic alteration of SF1 activity. There are several explanations for the different outcome on feeding behavior seen when constitutively deleting signaling pathways in the SF1-Cre mice (as opposed to acutely altering SF1 activity), including potential developmental effects, loss of function in extrahypothalamic sites, and changes in gene expression and other elements of neuronal function.

Our optogenetic data reveal a firing frequency-dependent switch from feeding to escape behavior, demonstrating the benefit of detailed phenotyping over a range of optogenetic stimulation frequencies. There are few studies employing a range of optogenetic stimulation frequencies, but these have also shown frequency-dependent phenotypic switches (Barbano et al., 2016; Belzung et al., 2014; Weitz et al., 2015). Studies to date utilizing optogenetics in SF1 neurons, and indeed AgRP neurons, have utilized stimulation protocols typically around 20 Hz (and often for several hours), not reflecting typical *in vivo* or *in vitro* firing patterns and limiting the ability to reveal behavioral changes caused by minor alterations in steady-state firing.

Our optogenetic data reveal that the switch from feeding to anxiety-related behavior is also sensitive to the energy storage status of mice. Mice with lower adipose tissue mass, therefore, tolerate a higher stimulation frequency before they display avoidance behavior when compared to mice with elevated fat stores. This is consistent with the idea that, in natural environments, mice with lower energy stores may take higher risks to secure

(E) Illustration of food-seeking and innate fear-inducing olfactory cue (TMT) avoidance behavioral task.
(F) Time spent at pots with combinations of food (F±) and TMT smell (S±) or all of the pots together.
(G) Food intake from F+S+ pot.
Data are expressed as mean values ± SEM. 1-way ANOVA with Sidak post hoc test was used for (G) and for different conditions in (F), while 2-way, RM, and Sidak were performed for (C) and (D) (*$p < 0.05$, **$p < 0.01$, ***$p < 0.001$, and ****$p < 0.0001$). See also Figure S5.

OPEN ACCESS
CellPress

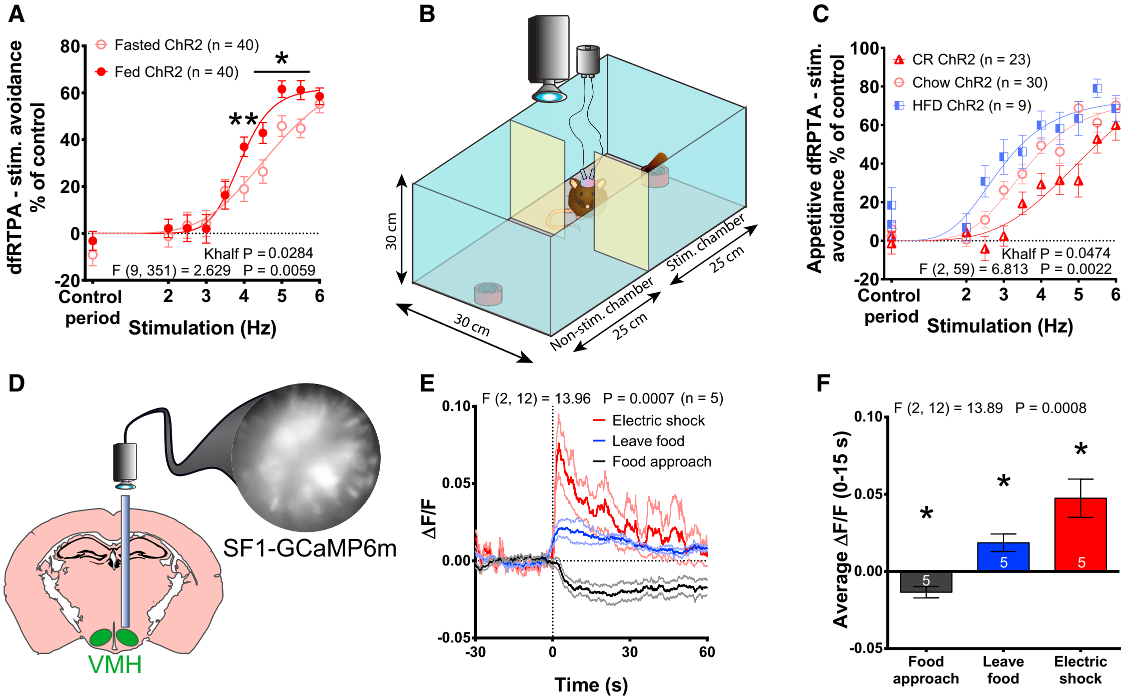

Figure 6. Nutrient and Energy Deficiency Blunts SF1 Neuron Stimulation-Induced Avoidance, while Activity Levels Encode Stress and Feeding States

(A) Avoidance of the stimulation chamber under a dfRTPA protocol during a fed-fasted crossover study in SF1-ChR2 animals, normalized to the respective controls.

(B) Illustration of appetitive dfRTPA task, designed to test the impact of nutrient deprivation on SF1-stimulation-induced avoidance in the presence of food.

(C) Avoidance of stimulation chamber in overnight-fasted calorie-restricted (CR), chow-fed (chow/fasted) and HFD-fed (HFD/fasted) SF1-ChR2 mice, normalized to the respective controls.

(D) Schematic of whole-field fluorescence recordings in SF1-GCaMP6m mice.

(E) Fluorescence changes in SF1-GCaMP6m mice after an electric shock, terminating feeding or approaching food.

(F) Averaged 15 s of changes in fluorescence from (E).

Data are expressed as mean values ± SEM. (A)–(C) were analyzed by 2-way ANOVA, RM measures, Sidak as well as by fitting graphs to non-linear regression. Symbols denote significance of post hoc tests but were omitted from (C) for visual clarity. 30-s baselines prior to the behaviors were used in (E). 1-way ANOVA was used for treatment effect in (E) and (F). 1-sample t test was used in (F) (*p < 0.05 and **p < 0.01). See also Figure S6.

food. In terms of potential mechanisms, previous studies have revealed that leptin alters SF1 neuron excitability. Deletion of the leptin receptor in these cells caused mild obesity, but this did not alter food intake, suggesting that leptin signaling per se may not mediate the effects we observed. It is possible that alterations in energy stores change the sensitivity of other nutrient-responsive circuits downstream of SF1 neurons, which in turn mediate nutrient-sensitive avoidance behavior.

SF1 Neurons Regulate Affective States Underlying Both Food Consummatory and Competing Behaviors to Create Activity-Dependent Feeding-Permissive or -Occlusive States

Homeostatic feeding is driven by hunger and appetite, and it requires integration of visceral and adiposity information regarding energy status. Food consumption, particularly in the wild, is, however, also dependent on the success of acquiring food, which can be reliant on underlying affective states to control the risks taken under various anxiogenic conditions. Therefore, the neurocircuits contributing to the control of nutrient intake, must also incorporate sensory, valence, arousal, motivational intensity, reward, and memory information to regulate a number of related behaviors, including foraging, risk-taking, and dealing with anxiogenic stimuli (Panksepp, 2010; Singh, 2014; Sweeney and Yang, 2017). Recent studies have explored this relationship between feeding and other related behaviors. For example, AgRP, lateral hypothalamic, and amygdalar neurons have important roles in the incorporation of associated emotional aspects into the regulation of feeding. Activation of AgRP neurons leads to voracious eating, and their activity has been shown to anticipate food while also establishing emotional states that promote feeding (reviewed in Sweeney and Yang, 2017). GABAergic and glutamatergic neurons in the lateral hypothalamus also regulate food intake by controlling rewarding aspects of feeding, while amygdalar neurocircuits have been implicated in control feeding in addition to regulating valence (reviewed in Sweeney and Yang, 2017).

SF1 neurons also represent an attractive population for regulating such aspects of feeding behavior, but recent studies using optogenetics and chemogenetics to investigate VMH and SF1

neuron function have mostly focused on specific defensive and fear behavioral outputs and did not analyze food intake. Thus, a male aggression locus has been identified in the ventrolateral subdivision of the VMH (vlVMH), and the activity of neurons in this region impacts on the competing behaviors of inter-male aggression and mating (Lee et al., 2014; Lin et al., 2011). Furthermore, optogenetic activation of SF1 neurons, which largely comprise the dorsomedial part of the VMH (dmVMH), drives different defensive behaviors in a range of contexts displaying features consistent with the establishment of a defensive emotional state (Kunwar et al., 2015). Likewise, separate optogenetic studies have suggested that the activation of different SF1 projections causes distinct defensive behaviors, such as immobility or avoidance (Wang et al., 2015). In addition, chemogenetic inhibition of SF1 neurons has been shown to suppress predator fear while inhibition of vlVMH neurons reduced social fear, suggesting partitioning of these behavioral outputs to different VMH neuronal populations (Silva et al., 2013, 2016). A recent study has demonstrated DREADD-mediated activation inhibits food intake, although the effects of suppressing SF1 activity upon feeding and the relationship to related behaviors were not explored (Coutinho et al., 2017).

We find that food intake elicited by the modulation of SF1 activity may result from both direct alterations in appetite and/or through changes in a range of feeding-permissive related behaviors. High-frequency stimulation of SF1 neurons is associated with states of high arousal, negative valence, and high motivational intensity, and this behavioral state is not consistent with feeding. Likewise, SF1 activity increased markedly under anxiogenic conditions. In contrast, pharmacogenetic and low-frequency optogenetic stimulation results in milder modulation of SF1 neurons and produces more nuanced changes in affective states. For example, we show that the inhibition of SF1 neurons produces increased feeding, specific interest in food, positive valence, and diminished anxiety while increasing locomotion and exploration. Together these conditions could be associated with an increased arousal state and wider cognitive scope and create an emotional state permissive for feeding to occur. On the other hand, low-frequency stimulation of SF1 neurons both suppressed food intake and lowered activity and exploration while increasing anxiety. Together these behaviors create a state that reduces feeding. Consistent with these observations, SF1 neurons showed decreased activity in freely behaving mice engaging with food and increased activity when mice moved away from food.

In tests examining the influence of SF1 neurons on feeding under naturalistic, stressful conditions that may occlude feeding, the inhibition of SF1 neurons created a behavioral state that was also more directed toward food intake. This low SF1 neuron activity, feeding-promoting behavioral state occurred irrespective of the negative stimulus used (e.g., open illuminated area, electric shocks, or innate fear-inducing smell), consistent with the idea that the VMH may regulate affective reactions to all sensory inputs and that these may be responsible for some of the appetitive behaviors (Grossman, 1966). However, we also observed that SF1-hM4Di mice have specific interest in food above that of non-edible objects, while 2-Hz optogenetically stimulated mice spent less time near the food, suggesting direct or indirect effects of SF1 neurons on appetite. It would thus appear that, most of the time, SF1 neurons are engaged in regulating feeding behavior and affective states that influence this but have the capacity to switch to high-firing frequency-dependent escape and defensive behavior when required, such as when faced by immediate predator threat or fighting with aggressive conspecifics. Our feeding and behavioral data reveal no phenotypic sexual dimorphism, suggesting some segregation in behavioral outputs and perhaps related to distinct neuronal populations (e.g., SF1- and ERα-expressing VMH neurons) being involved. Our data thus demonstrate that SF1 neurons constitute a behavioral gateway for feeding-permissive states, where inhibition of SF1 neurons shifts the balance between feeding and competing motivations toward consummatory behavior.

SF1 Neuron Integrative Function in the Context of Developing Model of Neurocircuits Controlling Feeding and Behavior

The SF1 circuitry can be incorporated into the developing model of brain integration of feeding and competing behavioral goals. VMH neurons directly synapse onto anorectic POMC neurons in a nutrient-sensitive manner (Sternson et al., 2005), providing a potential link between SF1 neurons and the melanocortin system, a key regulator of feeding and energy homeostasis. While not directly linked with AgRP neurons, SF1 neurons also share common projection sites with AgRP neurons, which presents an additional point of intersection between these systems (Betley et al., 2013; Cheung et al., 2013). Given that multiple hypothalamic neuronal populations may control both food intake and various associated behaviors, it is possible that there may be the potential for context-specific regulation of feeding depending on behavioral circumstances. Interestingly, sensory detection of food is associated with a reduction in AgRP neuron activity, akin to our findings in SF1 neurons when mice approach food. Interactions between these populations could thus constitute a key part of the neurocircuitry for behavioral selection between the competing motivations for food and safety, and future studies will be key for dissecting the precise relationships and hierarchy.

SF1 Neuron Function in the Context of Human Feeding and Anxiety

In the wild, feeding is a necessary set of actions required to procure nutrients and energy, but at the same time animals are often at their most vulnerable. Although in today's world food is easily accessible to most humans, our ancestors were frequently in danger from environmental factors, rivals, and predators when they ventured out from the safety of their dwellings to eat. Therefore, the regulatory systems have evolved to fine-tune and incorporate energy metabolism with emotional states and their behavioral outputs. These neurocircuits and signaling pathways that were necessary for survival have significant functional overlap, are complex and redundant, and are highly conserved to present-day humans (Nesse, 1998; Sweeney and Yang, 2017; Ulrich-Lai and Ryan, 2014). Therefore, in humans, mutations or defects in neural pathways causing obesity or metabolic disorders have a significant association with emotion-related disorders, such as anxiety and depression (Anderson et al.,

2001; Faith et al., 2002; Gariepy et al., 2010; Hryhorczuk et al., 2013; Stunkard et al., 2003). Importantly, while both metabolic disease such as obesity and emotional disorders are very common, there are few therapies that specifically target such diseases in part due to the common neuronal mechanisms regulating such processes. Thus, many drugs used to treat psychiatric disorders are associated with unwanted metabolic side effects, and many agents previously used to treat obesity displayed undesirable behavioral and psychological effects that have led to their withdrawal as treatments. Given the findings presented in this study, SF1 neurons are identified as an important part of the neurocircuitry regulating feeding and affective states, and, therefore, they may underpin some of these side effects and yet also provide new therapeutic opportunities for treating metabolic or emotional disorders.

EXPERIMENTAL PROCEDURES

Statistical Analysis and Data Reporting

Statistical comparisons were performed using parametric (un-)paired t tests or 1-way ANOVA, followed by a Sidak post hoc for single time-point data. 2-way ANOVA with repeated-measures (RM) and Sidak test or non-linear fitting was used for time course experiments. The precise statistical tests used are indicated in the figure legends, while F and p values, degrees of freedom, and sample size (n) are shown in the figures. Data are expressed as mean values ± SEM. Where significance is presented, p values are as follows: *$p < 0.05$, **$p < 0.01$, ***$p < 0.001$, and ****$p < 0.0001$.

Experiment Design

Male and female mice were used for most experiments, except for optogenetic experiments that used males only. For studies requiring surgery, adult mice aged 8–12 weeks old were used, and at least 3 weeks were allowed for recovery and virus expression before the start of the experiments. Power calculations for the number of animals required for every experiment were based on reported or known effect sizes and variation, in order to maximize chances of meaningful results without the unnecessary use of the experimental animals. Subject assignment to experimental groups was semi-random, and persons performing experiments, video tracking, scoring, and initial analysis were blinded to this and/or the treatment type where possible. Key physiology and behavior experiments were independently repeated. Raw data were collected in Excel and exported for statistical analyses to GraphPad Prism 6.0.

Animal Use Approval, Genetics, and Husbandry

All of the animal procedures conformed to the UK Animals (Scientific Procedures) Act 1986 as well as being approved by our institutional ethical review committee and by the UK Home Office. Findings and experiments described in this paper were designed and reported following the Animal Research: Reporting of *In Vivo* Experiments (ARRIVE) guidelines of animal experiment reporting (Kilkenny et al., 2010). The mouse line expressing Cre recombinase under *Nr5a1*-regulatory elements, referred to as SF1-Cre (Dhillon et al., 2006), was purchased from the Jackson Laboratory (012462) and backcrossed into C57BL/6 background for 4 generations. The Z/EG line was kindly donated by Dr. C. Lobe (Novak et al., 2000). Animals were housed in specific pathogen-free barrier facilities and maintained under a controlled environment (temperature 21°C–23°C, 12-hr light-dark cycle, lights on at 7:00 a.m.) with *ad libitum* access to food (RM3 chow diet, Special Diet Services) and water, unless stated otherwise. Animals were group-housed with up to 5 mice per cage for experimental studies, unless otherwise required and explicitly stated, e.g., for food intake studies, where mice were singly housed. Please see the Supplemental Experimental Procedures for details on genotyping.

Generation of Viral Vectors

Viruses used in this project included AAV1-Ef1a-DIO-hM3Dq-mCherry, AAV1-Ef1a-DIO-hM4Di-mCherry, AAV1-Ef1a-DIO-ChR2-mCherry, AAV1-Ef1a-DIO-EYFP, and AAV1-Syn-Flex-GCaMP6m-WPRE-SV40. The hM3Dq-mCherry coding sequence was cloned by Dr. A.I. Choudhury and Professor G. Milligan; the hM4Di sequence was commercially synthesized (GeneArt, Thermo Fisher Scientific), according to published data (Armbruster et al., 2007), and inserted into pAAV-EF1a-DIO-WPRE vectors instead of ChR2, which was gifted by Professor K. Deisseroth. Ef1a-DIO-EYFP was purchased from Addgene (Plasmid 27056, deposited by Professor K. Deisseroth) and commercially packaged in AAV serotype 2/1 vector consisting of the AAV2 ITR genomes and the AAV1 serotype capsid gene in Penn Vector Core, University of Pennsylvania. AAV1.Syn.Flex.GCaMP6m.WPRE.SV40 was purchased from Penn Vector Core (AV-1-PV2820). All other vectors were commercially packaged in the same manner in Vector Biolab, Philadelphia. All of the viruses below the titer of 1.2×10^{13} were used directly, while the titers above this were diluted in sterile PBS A, 5% glycerol (pH 7.2) down to 1.2×10^{13} genome copies.

Stereotaxic Surgery Procedure

Viruses used were stereotaxically targeted to the VMH (anteroposterior [AP], −1.35; mediolateral [ML], ± 0.45; dorsoventral [DV], −5.70). For optogenetic studies, custom-made optic fiber cannulae (200-μm diameter, 0.39-numerical aperture (NA) fiber with 1.25-mm ceramic ferrule; Thorlabs) were implanted at a 10-degree angle bilaterally above the VMH (AP, −1.35; ML, ± 1.50; DV, −5.10), of mice that had been previously injected with the AAV1-ChR2 in the same procedure. For Ca^{2+} imaging, a ProView gradient index (GRIN) lens (0.6-mm diameter, 7.3-mm length; Inscopix) was implanted ~200 μm above the VMH of AAV1-GCaMP6m-injected animals.

Optogenetic Stimulation Setup

Laser light sources used were ×1 473 nm, 50 mW (CL-473-050, CrystaLaser) and ×3 473 nm, 80 mW (STRADUS-473-80, Laser 2000). Light intensity of CrystaLaser devices was regulated mechanically, whereas light intensity of Stradus lasers was controlled by Vortran PC software. Laser activity patterns were transistor-transistor logic (TTL) governed by MC_Stimulus II software and two stimulus generators from Multichannel Systems (STG4004 and STG4008). Laser sources were connected to beam-splitting, collimated rotary joints (FRJ_1x2i_FC-2FC_0.22, Doric Lenses) via optic cables (diameter of 200 μm, 0.39 NA, M72L02; Thorlabs). The divided beam was fed into custom-length patch cords (fiber connector/physical connection [FC/PC] to 1.25-mm ceramic ferrule patch cord; Thorlabs) that was connected to the fiber implants on the animals via mating sleeves (1.25-mm diameter mating sleeve; Thorlabs).

Behavior Analysis

For DREADD studies, CNO (Key Organics) was administered via intraperitoneal (i.p.) injection 30 min prior to the assays (0.5 mg/kg for SF1-hM3Dq and 2.5 mg/kg for SF1-hM4Di animals) or in the drinking water at 50 mg/L. For optogenetic studies, 5-mW (2.8–1.0 mW/mm^2 in the VMH), 488-nm, 1-ms laser light stimulation was used (Aravanis et al., 2007; Yizhar et al., 2011). Animal behavior was filmed, video-tracked, and subsequently analyzed by the EthoVision XT (Noldus) software. Please see the Supplemental Experimental Procedures for more detail.

Prolonged Optogenetic Stimulation and Autonomic Function Assessment

Behavioral effects of long-term optogenetic stimulation were evaluated by continuously stimulating animals for 1 hr in a square open field at 2- or 5-Hz frequency and by 5-hr-long 2-Hz stimulation of mice in a home cage environment. Heart rate was measured after 2 hr of stimulation using ECGenie (Mouse Specifics), while the effects of optogenetic stimulation on brown adipose tissue temperature was measured using surgically implanted transponders (IPTT-300, Plexx).

In Vivo Ca^{2+} Dynamics

A miniature head-mounted camera (nVista, Inscopix) was used to perform microendoscopic imaging of Ca^{2+} dynamics in freely behaving, GRIN lens-implanted SF1-GCaMP6m animals. The effects of food were analyzed by placing overnight-fasted animals in a 2-chamber arena with empty pots in the opposite corners. After 10 min of exploration, food and a non-edible object were placed

in the different pots for an additional 20 min. To enable accurate behavioral correlation with neuronal activity, freely behaving animals were tracked with Ethovision XT software. When a specific behavior was detected (e.g., entrance into the feeding zone), TTL signals were sent via an external module into the data acquisition (DAQ) box, and timestamps were subsequently recorded in the nVista acquisition software. Electric shocks (0.4 mA) in the fear-conditioning box (Med Associates) were given at predefined times. Data were processed using Mosaic software (Inscopix) and analyzed in Excel. 30 s of recording before the onset of behavior/shock was used as a baseline for the calculations of the change in fluorescence for every time point.

Ex Vivo Electrophysiology

Slice electrophysiology was performed essentially as previously described (Smith et al., 2015). In brief, brains of SF1-Cre transgenic mice transfected with EYFP, hM3Dq, hM4Di, or ChR2 virus were rapidly transferred to an ice-cold slicing solution containing 2.5 mM KCl, 1.25 mM NaH_2PO_4, 28 mM $NaHCO_3$, 0.5 mM $CaCl_2$, 7 mM $MgCl_2$, 7 mM D-glucose, and 235 mM sucrose, and they were equilibrated with 95% O_2 5% CO_2 to give a pH of 7.4. The 350-μm coronal brain slices were prepared using a Vibratome Series 1000. Slices containing the VMH were kept at room temperature in a normal external solution, containing 125 mM NaCl, 2.5 mM KCl, 1.25 mM NaH_2P_4, 25 mM $NaHCO_3$, 2 mM $CaCl_2$, 1 mM $MgCl_2$, 10 mM D-glucose, 15 mM D-mannitol, equilibrated with 95% O_2 5% CO_2 (pH 7.4). For whole-cell recordings, slices were continuously perfused with normal external solution at a rate of 5–10 mL/min. Neurons were identified using epifluorescence and differential interference contrast optics, using an upright Slicescope (Scientifica) microscope. Borosilicate patch pipettes (5–8 MΩ) were filled with an internal solution containing 130 mM potassium gluconate, 10 mM KCl, 0.5 mM EGTA, 10 mM HEPES, 1 mM NaCl, 0.28 mM $CaCl_2$, 3 mM $MgCl_2$, 3 mM KATP, 0.3 mM Tris-GTP, and 14 mM phosphocreatine (pH 7.2). Data were digitally recorded and stored for offline analysis. We examined changes in input resistance by monitoring membrane potential responses to tonic-negative, rectangular current pulses (5–20 pA, 200 ms, 0.05 Hz) or by current-voltage relations (−80 to +30 pA, 200 ms) injected via the recording electrode. Application of drugs or CNO was made via the bath perfusion system at the concentrations indicated.

In Vivo Electrophysiology

Mice were anesthetized with isoflurane in an induction chamber. The animal was then i.p. injected with urethane (Ethyl carbamate, 25% w/v, dose of 1.5 g/kg) and glycopyrrolate (0.01 mg/kg) and placed into a stereotaxic frame. It was monitored for 20 min to judge the depth of anesthesia (breathing rate and pinch reflex). Anesthesia was topped up if required by 10% of the initial dose, but with at least 20-min periods of rest. Bupivacaine (2.5 mg/mL) was administered locally prior to the surgery. The animal was kept warm with an insulation cover, and core body temperature was monitored with a thermometer probe. The skin over the skull was removed and the skull was cleaned with saline. A craniotomy was performed above the VMH by drilling a rectangle in the skull and lifting the bone with tweezers. 0.45% saline was used to wash the surface of the brain. The electric circuit was grounded into the neck muscle posterior of the skull. A glass microelectrode, with a tip diameter of 1–1.5 μm, resistance of 15–25 MΩ, and filled with 1.5% neurobiotin in 0.5 M NaCl, was slowly lowered (1–10 μm/min) into the brain toward the target area.

Coordinates used for recordings in the VMH were as follows: AP, −1.3 to −1.6; DV, −5.25 to −5.75; and ML, ± 0.45 mm. When the electrode reached the target area, the movement speed was decreased. Recording began when a clearly isolated single unit was detected. Neuronal activity was typically measured for at least 10 min before the animal was i.p. injected with CNO (doses used for *in vivo* experiments: 0.5 mg/kg for hM3Dq and 2.5 mg/kg for hM4Di). In some recordings, an additional high dose (50 mg/kg) of CNO was injected to confirm the responsiveness of the recorded neurons. Recordings were AC-coupled, amplified (×1,000), filtered (0.3–5 kHz), and acquired with Spike 2 software on a PC. For statistical analysis, action potential events were assigned into 20-s bins. Periods of 5 min before and 15 min after CNO injection were compared statistically. Mean firing frequency was calculated as an average frequency over the 5-min periods. Peak firing frequency was the frequency of a 20-s bin with the highest firing frequency during the 5-min analysis period. After the recording, some neurons were selectively labeled by the juxtacellular technique with biotin that involved positive current-pulse application through the microelectrode (200 ms, 2.5 Hz, 1–5 nA). Changes in the frequency were normalized between neurons using *Z* scores, where the population mean and SD were estimated from the initial 5 min of recording.

SUPPLEMENTAL INFORMATION

Supplemental Information includes Supplemental Experimental Procedures and six figures and can be found with this article online at https://doi.org/10.1016/j.celrep.2017.11.089.

ACKNOWLEDGMENTS

We are grateful to Eleanor Sandhu, Steve Millership, Suchira Gallage, Olga Boruc, and Megan Woodberry for technical help and advice. This work was supported by funding from the Biotechnology and Biological Sciences Research Council to G.M. (BB/K019864/1) and from the Medical Research Council to D.J.W. (MC-A654-5QB40) and M.A.U. (MC-A654-5QB70).

AUTHOR CONTRIBUTIONS

Conceptualization, P.V., E.E.I., M.A.S., and D.J.W.; Methodology, P.V., E.E.I., M.A.S., A.I.C., and D.J.W.; Investigation, P.V., E.E.I., M.A.S., J.A.G., D.G.H., S.M.A.P., M.R.P.P., L.K., A.B.P.F., M.A.U., and D.J.W.; Resources, E.A.-C., G.M., A.S., M.A.U., and D.J.W.; Writing – Original Draft, P.V. and D.J.W.; Writing, Review & Editing, P.V., E.E.I., M.A.S., and D.J.W.; Funding Acquisition, M.A.U., G.M., and D.J.W.; Visualization, P.V.; Supervision, D.J.W.

DECLARATION OF INTERESTS

The authors declare no competing interests.

Received: July 26, 2017
Revised: October 18, 2017
Accepted: November 27, 2017
Published: December 19, 2017

REFERENCES

Anderson, R.J., Freedland, K.E., Clouse, R.E., and Lustman, P.J. (2001). The prevalence of comorbid depression in adults with diabetes: a meta-analysis. Diabetes Care *24*, 1069–1078.

Aravanis, A.M., Wang, L.-P., Zhang, F., Meltzer, L.A., Mogri, M.Z., Schneider, M.B., and Deisseroth, K. (2007). An optical neural interface: in vivo control of rodent motor cortex with integrated fiberoptic and optogenetic technology. J. Neural Eng. *4*, S143–S156.

Armbruster, B.N., Li, X., Pausch, M.H., Herlitze, S., and Roth, B.L. (2007). Evolving the lock to fit the key to create a family of G protein-coupled receptors potently activated by an inert ligand. Proc. Natl. Acad. Sci. USA *104*, 5163–5168.

Barbano, M.F., Wang, H.-L., Morales, M., and Wise, R.A. (2016). Feeding and Reward Are Differentially Induced by Activating GABAergic Lateral Hypothalamic Projections to VTA. J. Neurosci. *36*, 2975–2985.

Belzung, C., Turiault, M., and Griebel, G. (2014). Optogenetics to study the circuits of fear- and depression-like behaviors: a critical analysis. Pharmacol. Biochem. Behav. *122*, 144–157.

Betley, J.N., Cao, Z.F.H., Ritola, K.D., and Sternson, S.M. (2013). Parallel, redundant circuit organization for homeostatic control of feeding behavior. Cell *155*, 1337–1350.

Boden, P., Ashford, M.L., and Treherne, J.M. (1989). Actions of sulfonylureas on neurones of rat ventromedial hypothalamus in vitro. Br. J. Pharmacol. *98* (*Suppl*), 830P.

Cardinal, P., André, C., Quarta, C., Bellocchio, L., Clark, S., Elie, M., Leste-Lasserre, T., Maitre, M., Gonzales, D., Cannich, A., et al. (2014). CB1 cannabinoid

receptor in SF1-expressing neurons of the ventromedial hypothalamus determines metabolic responses to diet and leptin. Mol. Metab. *3*, 705–716.

Cheung, C.C., Kurrasch, D.M., Liang, J.K., and Ingraham, H.A. (2013). Genetic labeling of steroidogenic factor-1 (SF-1) neurons in mice reveals ventromedial nucleus of the hypothalamus (VMH) circuitry beginning at neurogenesis and development of a separate non-SF-1 neuronal cluster in the ventrolateral VMH. J. Comp. Neurol. *521*, 1268–1288.

Cheung, C.C., Krause, W.C., Edwards, R.H., Yang, C.F., Shah, N.M., Hnasko, T.S., and Ingraham, H.A. (2015). Sex-dependent changes in metabolism and behavior, as well as reduced anxiety after eliminating ventromedial hypothalamus excitatory output. Mol. Metab. *4*, 857–866.

Coutinho, E.A., Okamoto, S., Ishikawa, A.W., Yokota, S., Wada, N., Hirabayashi, T., Saito, K., Sato, T., Takagi, K., Wang, C.-C., et al. (2017). Activation of SF1 Neurons in the Ventromedial Hypothalamus by DREADD Technology Increases Insulin Sensitivity in Peripheral Tissues. Diabetes *66*, 2372–2386.

Dhillon, H., Zigman, J.M., Ye, C., Lee, C.E., McGovern, R.A., Tang, V., Kenny, C.D., Christiansen, L.M., White, R.D., Edelstein, E.A., et al. (2006). Leptin directly activates SF1 neurons in the VMH, and this action by leptin is required for normal body-weight homeostasis. Neuron *49*, 191–203.

Faith, M.S., Matz, P.E., and Jorge, M.A. (2002). Obesity-depression associations in the population. J. Psychosom. Res. *53*, 935–942.

Gariepy, G., Nitka, D., and Schmitz, N. (2010). The association between obesity and anxiety disorders in the population: a systematic review and meta-analysis. Int. J. Obes. *34*, 407–419.

Grossman, S.P. (1966). The VMH: A center for affective reactions, satiety, or both? Physiol. Behav. *1*, 1–10.

Hryhorczuk, C., Sharma, S., and Fulton, S.E. (2013). Metabolic disturbances connecting obesity and depression. Front. Neurosci. *7*, 177.

Kilkenny, C., Browne, W.J., Cuthill, I.C., Emerson, M., and Altman, D.G. (2010). Improving bioscience research reporting: the ARRIVE guidelines for reporting animal research. PLoS Biol. *8*, e1000412.

Kim, K.W., Jo, Y.-H., Zhao, L., Stallings, N.R., Chua, S.C., Jr., and Parker, K.L. (2008). Steroidogenic factor 1 regulates expression of the cannabinoid receptor 1 in the ventromedial hypothalamic nucleus. Mol. Endocrinol. *22*, 1950–1961.

Kim, K.W., Donato, J., Jr., Berglund, E.D., Choi, Y.-H., Kohno, D., Elias, C.F., Depinho, R.A., and Elmquist, J.K. (2012). FOXO1 in the ventromedial hypothalamus regulates energy balance. J. Clin. Invest. *122*, 2578–2589.

King, B.M. (2006). The rise, fall, and resurrection of the ventromedial hypothalamus in the regulation of feeding behavior and body weight. Physiol. Behav. *87*, 221–244.

Klöckener, T., Hess, S., Belgardt, B.F., Paeger, L., Verhagen, L.A., Husch, A., Sohn, J.W., Hampel, B., Dhillon, H., Zigman, J.M., et al. (2011). High-fat feeding promotes obesity via insulin receptor/PI3K-dependent inhibition of SF-1 VMH neurons. Nat. Neurosci. *14*, 911–918.

Kunwar, P.S., Zelikowsky, M., Remedios, R., Cai, H., Yilmaz, M., Meister, M., and Anderson, D.J. (2015). Ventromedial hypothalamic neurons control a defensive emotion state. eLife *4*, 1–30.

Lee, H., Kim, D.-W., Remedios, R., Anthony, T.E., Chang, A., Madisen, L., Zeng, H., and Anderson, D.J. (2014). Scalable control of mounting and attack by Esr1+ neurons in the ventromedial hypothalamus. Nature *509*, 627–632.

Lin, D., Boyle, M.P., Dollar, P., Lee, H., Lein, E.S., Perona, P., and Anderson, D.J. (2011). Functional identification of an aggression locus in the mouse hypothalamus. Nature *470*, 221–226.

Nesse, R. (1998). Emotional disorders in evolutionary perspective. Br. J. Med. Psychol. *71*, 397–415.

Novak, A., Guo, C., Yang, W., Nagy, A., and Lobe, C.G. (2000). Z/EG, a double reporter mouse line that expresses enhanced green fluorescent protein upon Cre-mediated excision. Genesis *28*, 147–155.

Panksepp, J. (2010). Affective neuroscience of the emotional BrainMind: evolutionary perspectives and implications for understanding depression. Dialogues Clin. Neurosci. *12*, 533–545.

Ramadori, G., Fujikawa, T., Anderson, J., Berglund, E.D., Frazao, R., Michán, S., Vianna, C.R., Sinclair, D.A., Elias, C.F., and Coppari, R. (2011). SIRT1 deacetylase in SF1 neurons protects against metabolic imbalance. Cell Metab. *14*, 301–312.

Silva, B.A., Mattucci, C., Krzywkowski, P., Murana, E., Illarionova, A., Grinevich, V., Canteras, N.S., Ragozzino, D., and Gross, C.T. (2013). Independent hypothalamic circuits for social and predator fear. Nat. Neurosci. *16*, 1731–1733.

Silva, B.A., Mattucci, C., Krzywkowski, P., Cuozzo, R., Carbonari, L., and Gross, C.T. (2016). The ventromedial hypothalamus mediates predator fear memory. Eur. J. Neurosci. *43*, 1431–1439.

Singh, M. (2014). Mood, food, and obesity. Front. Psychol. *5*, 925.

Smith, M.A., Katsouri, L., Irvine, E.E., Hankir, M.K., Pedroni, S.M.A., Voshol, P.J., Gordon, M.W., Choudhury, A.I., Woods, A., Vidal-Puig, A., et al. (2015). Ribosomal S6K1 in POMC and AgRP Neurons Regulates Glucose Homeostasis but Not Feeding Behavior in Mice. Cell Rep. *11*, 335–343.

Sternson, S.M., Shepherd, G.M.G., and Friedman, J.M. (2005). Topographic mapping of VMH–> arcuate nucleus microcircuits and their reorganization by fasting. Nat. Neurosci. *8*, 1356–1363.

Stunkard, A.J., Faith, M.S., and Allison, K.C. (2003). Depression and obesity. Biol. Psychiatry *54*, 330–337.

Sweeney, P., and Yang, Y. (2017). Neural Circuit Mechanisms Underlying Emotional Regulation of Homeostatic Feeding. Trends Endocrinol. Metab. *28*, 437–448.

Ulrich-Lai, Y.M., and Ryan, K.K. (2014). Neuroendocrine circuits governing energy balance and stress regulation: functional overlap and therapeutic implications. Cell Metab. *19*, 910–925.

Wang, L., Chen, I.Z., and Lin, D. (2015). Collateral pathways from the ventromedial hypothalamus mediate defensive behaviors. Neuron *85*, 1344–1358.

Weitz, A.J., Fang, Z., Lee, H.J., Fisher, R.S., Smith, W.C., Choy, M., Liu, J., Lin, P., Rosenberg, M., and Lee, J.H. (2015). Optogenetic fMRI reveals distinct, frequency-dependent networks recruited by dorsal and intermediate hippocampus stimulations. Neuroimage *107*, 229–241.

Xu, Y., Hill, J.W., Fukuda, M., Gautron, L., Sohn, J.-W., Kim, K.-W., Lee, C.E., Choi, M.J., Lauzon, D.A., Dhillon, H., et al. (2010). PI3K signaling in the ventromedial hypothalamic nucleus is required for normal energy homeostasis. Cell Metab. *12*, 88–95.

Xu, Y., Nedungadi, T.P., Zhu, L., Sobhani, N., Irani, B.G., Davis, K.E., Zhang, X., Zou, F., Gent, L.M., Hahner, L.D., et al. (2011). Distinct hypothalamic neurons mediate estrogenic effects on energy homeostasis and reproduction. Cell Metab. *14*, 453–465.

Yizhar, O., Fenno, L.E., Davidson, T.J., Mogri, M., and Deisseroth, K. (2011). Optogenetics in neural systems. Neuron *71*, 9–34.

Cell Reports, Volume *21*

Supplemental Information

Modulation of SF1 Neuron Activity

Coordinately Regulates Both Feeding Behavior

and Associated Emotional States

Paulius Viskaitis, Elaine E. Irvine, Mark A. Smith, Agharul I. Choudhury, Elisa Alvarez-Curto, Justyna A. Glegola, Darran G. Hardy, Silvia M.A. Pedroni, Maria R. Paiva Pessoa, Anushka B.P. Fernando, Loukia Katsouri, Alessandro Sardini, Mark A. Ungless, Graeme Milligan, and Dominic J. Withers

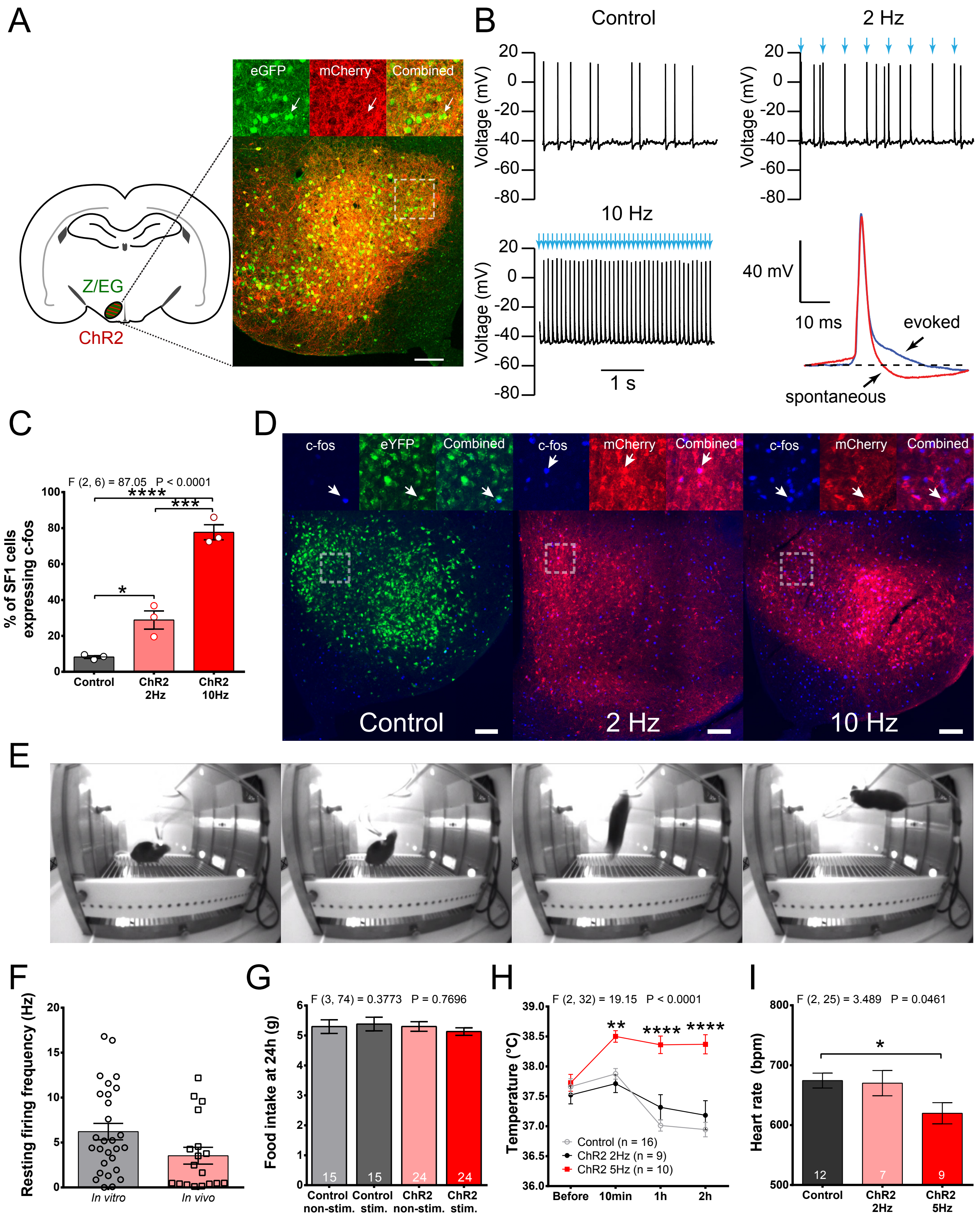

A
eGFP
mCherry
Combined
Z/EG
ChR2
B
Control
2 Hz
10 Hz
Voltage (mV)
20
0
-20
-40
-60
-80
1 s
40 mV
10 ms
evoked
spontaneous
C
F (2, 6) = 87.05 P < 0.0001
% of SF1 cells expressing c-fos
Control
ChR2 2Hz
ChR2 10Hz
D
c-fos
eYFP
Combined
mCherry
Control
2 Hz
10 Hz
E
F
Resting firing frequency (Hz)
In vitro
In vivo
G
F (3, 74) = 0.3773 P = 0.7696
Food intake at 24h (g)
Control non-stim.
Control stim.
ChR2 non-stim.
ChR2 stim.
H
F (2, 32) = 19.15 P < 0.0001
Temperature (°C)
Control (n = 16)
ChR2 2Hz (n = 9)
ChR2 5Hz (n = 10)
Before
10min
1h
2h
I
F (2, 25) = 3.489 P = 0.0461
Heart rate (bpm)
Control
ChR2 2Hz
ChR2 5Hz

Figure S1. Related to Figure 1.

(**A**) Illustration of the anatomic location of the VMH and microscope image showing co-expression of ChR2-mCherry with eGFP in AAV1-DIO-ChR2-mCherry injected SF1-Z/EG mice. Contrast increased for presentation purposes. (**B**) Slice electrophysiology traces during control, 2 Hz and 10 Hz stimulation conditions in current clamp settings, together with representative spontaneous and optogenetically-induced action potentials. (**C**) Quantification of c-fos expression 90 min following the initiation of optogenetic stimulation at 2 or 10 Hz. (**D**) Representative tiled images of the experiment in **C**. Contrast increased uniformly across groups for presentation purposes. (**E**) Example of a 20 Hz frequency optogenetic stimulation-induced escape behaviour in an operant box. (**F**) Steady state firing frequency of SF1 neurons in *ex vivo* (n = 28) or *in vivo* (n = 18) studies. (**G**) 24 h cumulative food intake of SF1-ChR2 mice following 5 h of optogenetic stimulation during a fast-refeed (sample size displayed on the graph). (**H**) Body temperature before or after 10 min, 1 h and 2 h of 2 or 5 Hz optogenetic stimulation. (**I**) Heart rate after continuous 2 h optogenetic stimulation. Scale bars in **A** and **D** are 100 μm. 1-way ANOVA, followed by Sidak post-hoc was performed, with results displayed on all figures except **F** and **H** that were analysed by t-test and 2-way ANOVA respectively. Data presented as mean ± SEM. * $p < 0.05$, ** $p < 0.01$, *** $p < 0.001$, **** $p < 0.0001$.

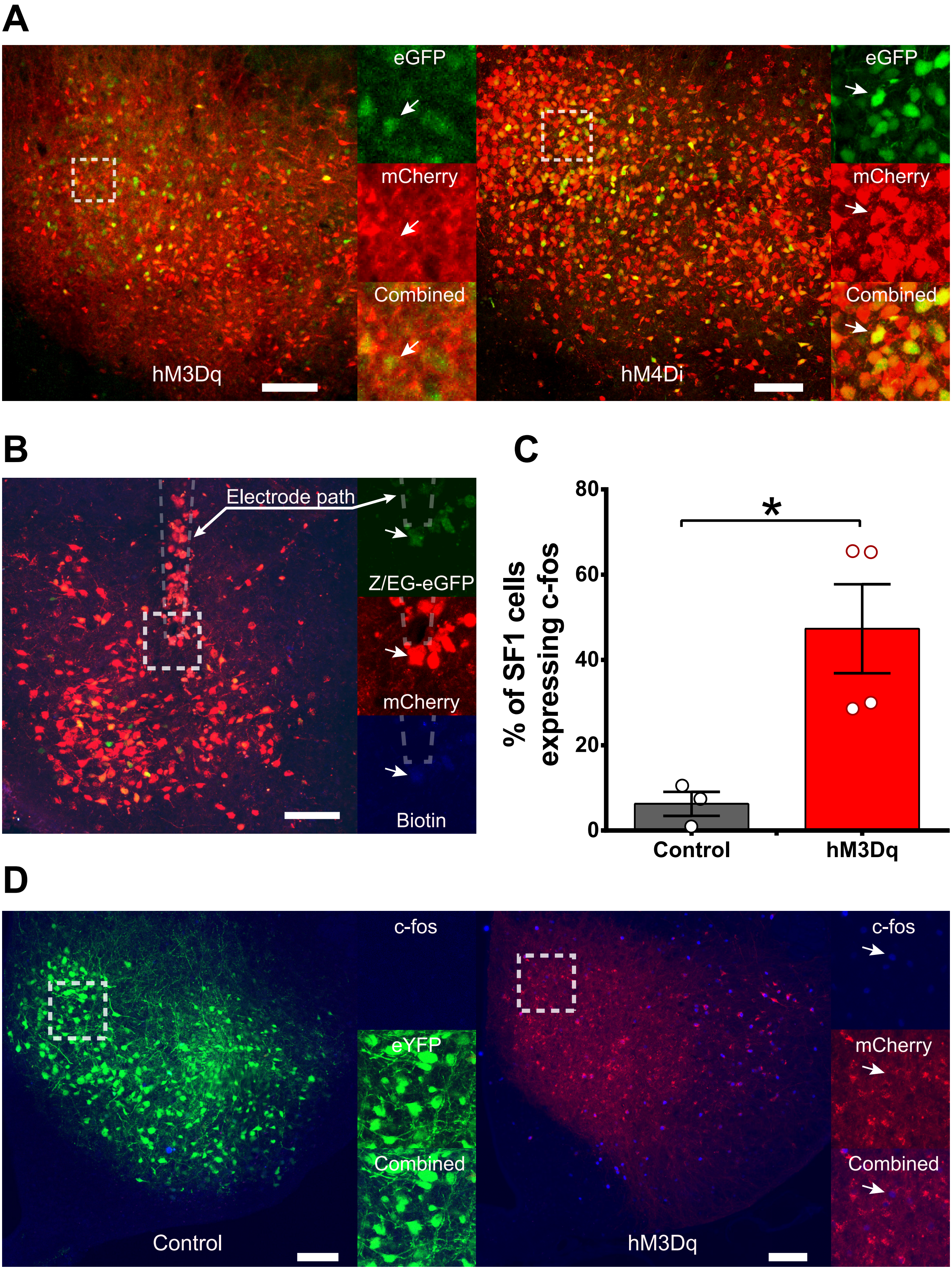
A
eGFP
mCherry
Combined
hM3Dq
eGFP
mCherry
Combined
hM4Di
B
Electrode path
Z/EG-eGFP
mCherry
Biotin
C
% of SF1 cells expressing c-fos
80
60
40
20
0
*
Control
hM3Dq
D
c-fos
eYFP
Combined
Control
c-fos
mCherry
Combined
hM3Dq

Figure S2. Related to Figure 2.
(**A**) Tiled images demonstrating co-expression of mCherry with eGFP in AAV1-DIO-hM3Dq-mCherry or AAV1-DIO-hM4Di-mCherry injected SF1-Z/EG mice. Contrast increased uniformly across groups for representation purposes. (**B**) An example of a resultant electrode path and biotin staining after *in vivo* electrophysiology recordings. (**C**) Proportion of SF1 neurons expressing c-fos (control n = 3; hM3Dq n = 4). (**D**) Representative tiled images of c-fos immunostaining in the VMH of control and SF1-hM3Dq animals 90 min after CNO administration. Contrast increased identically for all images for representation purposes. Scale bars in **A**, **B** and **D** are 100 μm. **C** analysed by a paired t-test. Data presented as mean ± SEM. * $p < 0.05$.

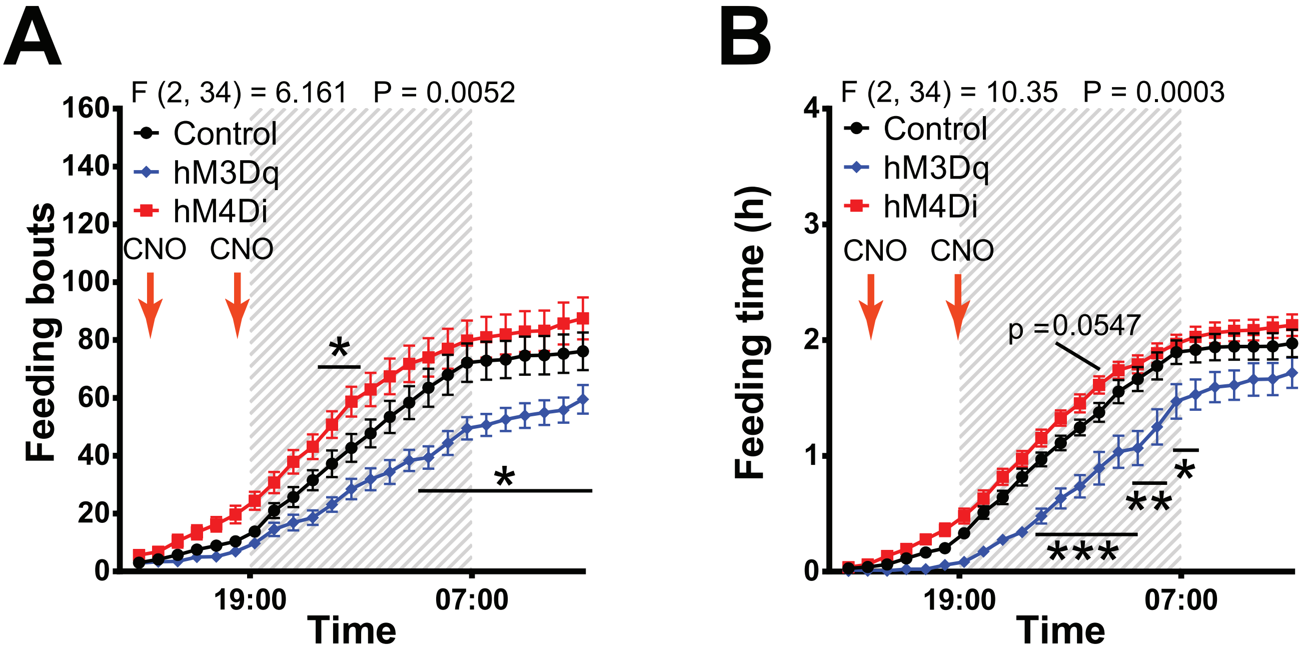

Figure S3. Related to Figure 3.
(**A**) Cumulative numbers of feeding bouts detected in the BioDAQ feeding system (control n = 13, hM3Dq n = 8, hM4Di, n = 16). (**B**) Same experiment as in **A**, but cumulative time spent feeding shown. Analysed by 2-way repeated measures ANOVA, Sidak post-hoc. Data presented as mean ± SEM. * $p < 0.05$, ** $p < 0.01$, *** $p < 0.001$.

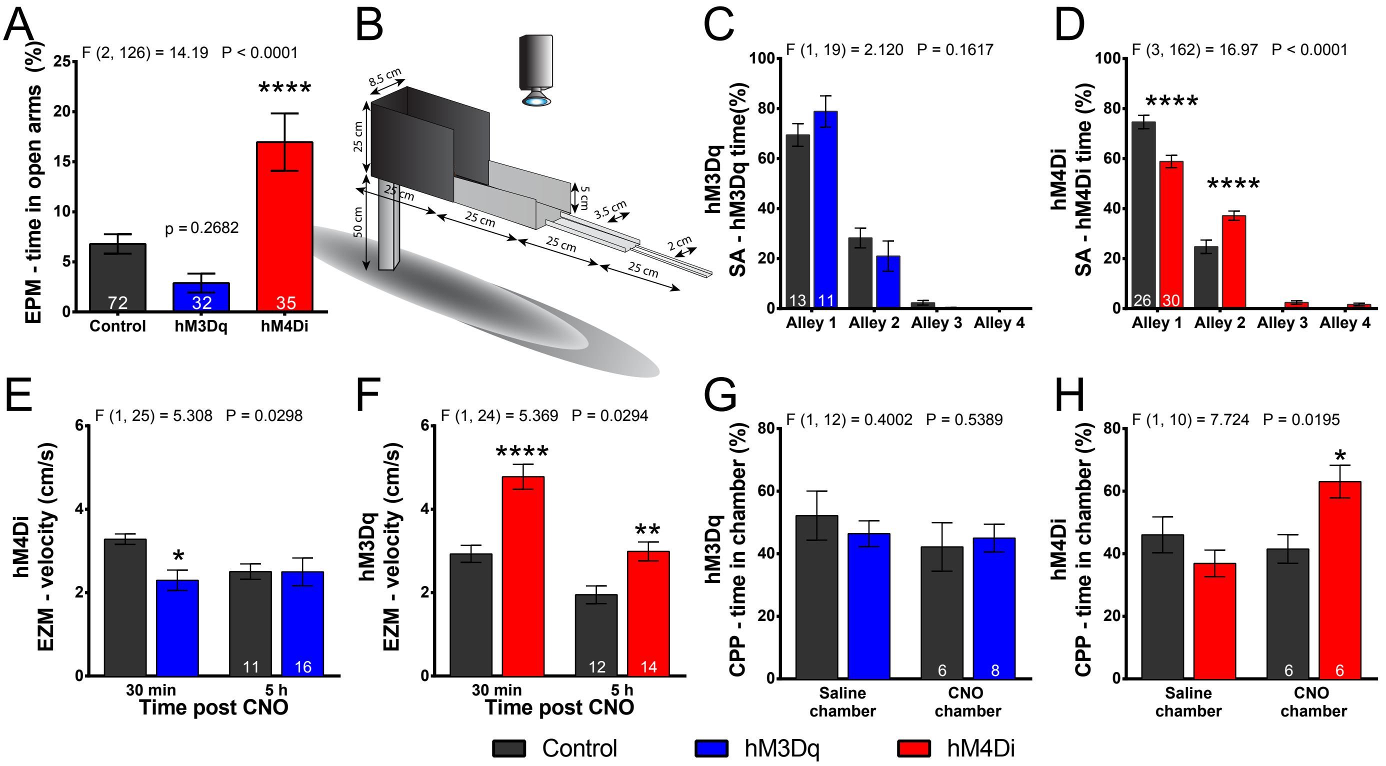

Figure S4. Related to Figure 4.

(**A**) Time spent in open areas of elevated plus maze (EPM). (**B**) Depiction of the successive alleys (SA) arena. Time spent per individual alleys of SA task by SF1-hM3Dq (**C**) and SF1-hM4Di mice (**D**). Velocity of SF1-hM3Dq (**E**) and SF1-hM4Di mice during EZM (**F**). (**G**) Conditioned place preference for chambers in which control or SF1-hM3Dq animals received either vehicle or CNO IP injections. (**H**) Identical to **G**, but results for SF1-hM4Di mice. Sample sizes are displayed on the appropriate columns. **A** analysed by 1-way ANOVA and Sidak post-hoc, while the rest of the studies were analysed by 2-way repeated measures ANOVA, Sidak post-hoc. Data presented as mean ± SEM. * $p < 0.05$, ** $p < 0.01$, **** $p < 0.0001$.

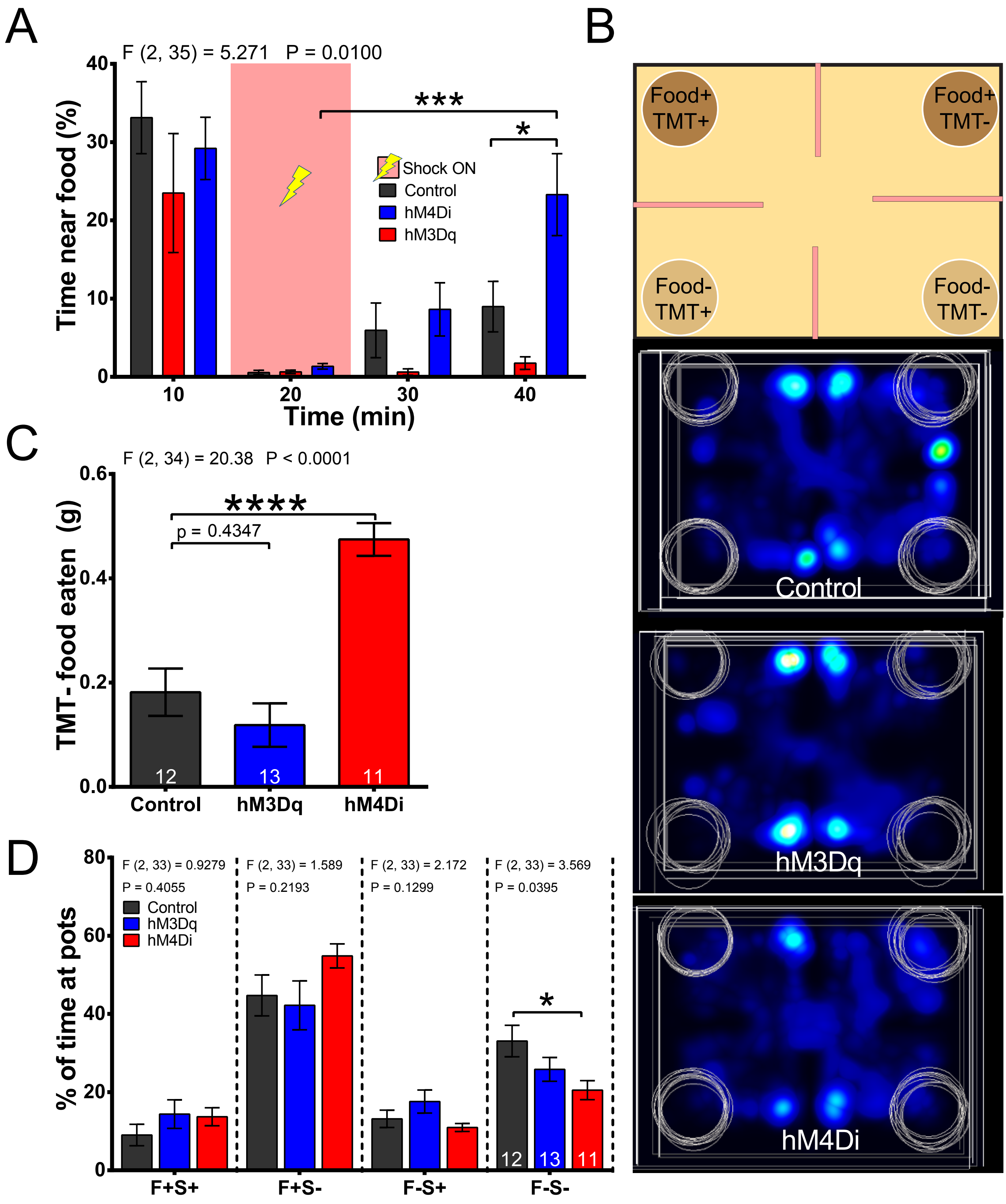

Figure S5. Related to Figure 5.

(**A**) Time spent in the feeding area during the negative feeding conditioning assay (control n = 7; hM3Dq n = 7; hM4Di n = 8). (**B**) Area subdivision of the TMT avoidance assay depicted in Figure 5E and heat-maps of place preference of control, SF1-hM3Dq and SF1-hM4Di mice during this task. (**C**) Food intake from TMT unscented pot. (**D**) Time spent at individual pots divided by the total time spent at all of the pots by that mouse. **A** was analysed by 2-way repeated measures ANOVA, Sidak post-hoc, while **C** and individual pots in **D** were analysed by 1-way ANOVA and Sidak post-hoc tests. Sample sizes indicated on the panels. Data presented as mean ± SEM. * $p < 0.05$, *** $p < 0.001$, **** $p < 0.0001$.

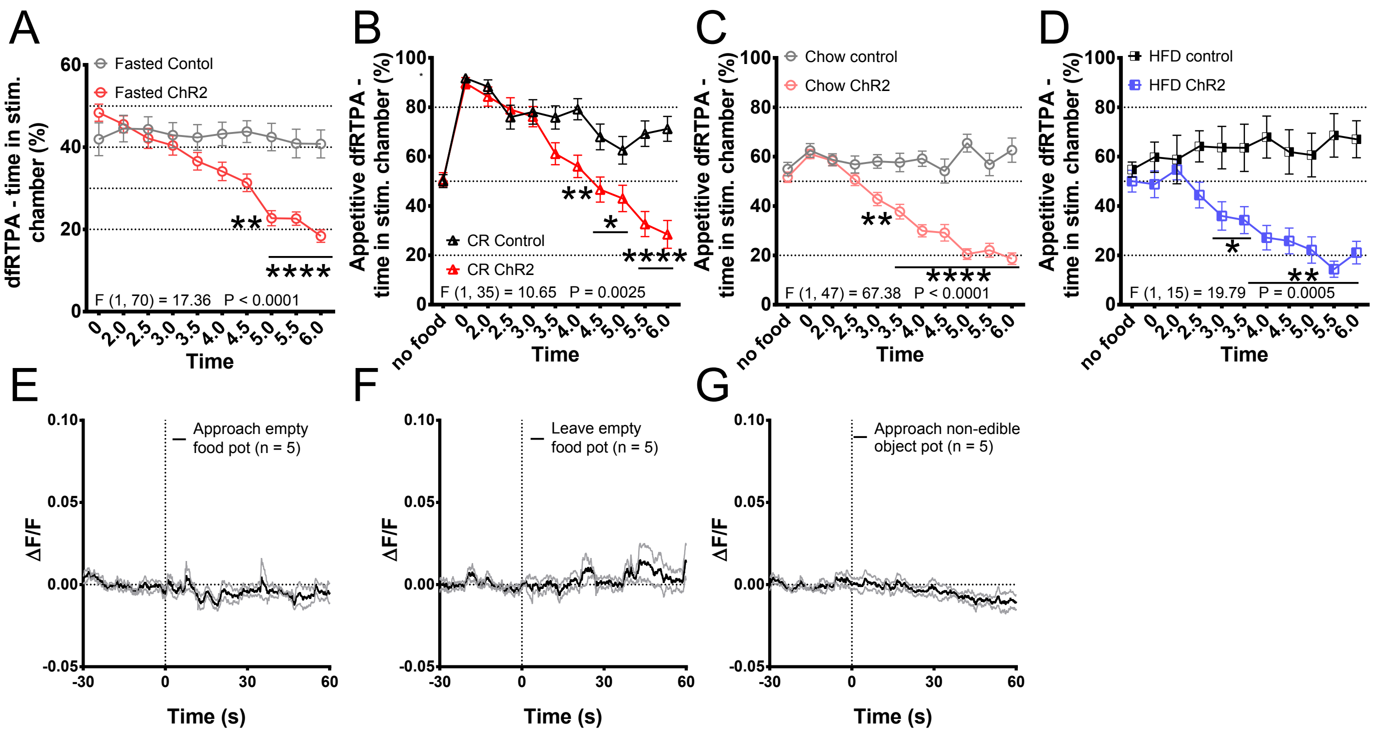

Figure S6. Related to Figure 6.
(**A**) Time spent in stimulation chamber by fasted control and SF1-ChR2 mice during the dfRTPA task (control n = 32; ChR2 = 40). (**B**) Time spent in stimulation chamber by overnight fasted, calorie restricted (CR) control and SF1-ChR2 animals during appetitive dfRTPA assay (control n = 14; ChR2 = 23). (**C**) Same as in **B**, but for chow fed mice (control n = 19; ChR2 = 30). (**D**) Identical to **B** and **C**, but in high-fat fed animals (control n = 8; ChR2 = 9). (**E**) Fluorescence changes in SF1-GCaMP6m mice aligned to approach of an empty pot, later in which food was placed. (**F**-**G**) Fluorescence changes when approaching or leaving an empty food pot, or when approaching a non-edible object. **A**-**D** analysis performed using 2-way repeated measures ANOVA, Sidak post-hoc. 30 s baselines prior to the behaviors were used in **E**-**G**. Data presented as mean ± SEM. * $p < 0.05$, ** $p < 0.01$, **** $p < 0.0001$.

Extended experimental procedures

Key resources table

Reagent or Resource	Source	Identifier
Antibodies		
Anti c-fos	Cell Signaling Technologies	#2250
Anti-rabbit IgG (H+L), conjugated with Alexa Fluor 647	Molecular Probes	A-21443
Experimental Models: Mouse lines		
SF1-Cre	Jackson Laboratory	012462
Z/EG	(Novak et al., 2000)	N/A
Chemicals (Pharmacological compounds)		
Clozapine N-oxide	Key Organics	N/A
Recombinant DNA		
AAV1-Ef1a-DIO-hM3Dq-mCherry	Dr A.I. Choudhury in collaboration with Prof G. Milligan	N/A
AAV1-Ef1a-DIO-hM4Di-mCherry	GeneArt, Thermo Fisher (Armbruster et al., 2007)	N/A
AAV1-Ef1a-DIO-ChR2-mCherry	Prof K. Deisseroth	N/A
AAV1-Ef1a-DIO-EYFP	Addgene	Plasmid #27056
AAV1.Syn.Flex.GCaMP6m.WPRE.SV40	Penn Vector Core	#2820
Sequence-Based Reagents		
NR5A1 forward 5' – CTGAGCTGCAGCGCAGGGACAT – 3'	(Dhillon et al., 2006)	N/A
NR5A1 reverse 5' – TGCGAACCTCATCACTCGTTGCAT – 3'	(Dhillon et al., 2006)	N/A
Z/eg forward 5' – AAGTTCATCTGCACCACCG – 3'	(Novak et al., 2000)	N/A

Z/eg reverse 5' – TCCTTGAAGAAGATGGTGCG – 3'	(Novak et al., 2000)	N/A
Optogenetic and imaging reagents		
Laser CL-473-050	CrystaLaser	CL-473-050
Laser STRADUS-473-80	Laser 2000	473-80
Stimulus generators	Multichannel Systems	STG4004 and STG4008
Beam-splitting, rotary joints	Doric Lenses	FRJ_1x2i_FC-2FC_0.22
Optic cables (200 µm, 0.39 NA, M72L02)	Thorlabs	M72L02
FC/PC to 1.25mm ferrule patch cords	Thorlabs	Custom
Mating sleeves	Thorlabs	AFAF1
nVista microscope	Inscopix	N/A
Baseplate	Inscopix	1050-002192
Baseplate cover	Inscopix	1050-002193
ProView™ Lens Probe; 0.6mm diameter, ~7.3mm length	Inscopix	100-000586
I/O box with XCAB-45BN cable	Noldus	N/A
Software and Algorithms		
ImageJ	https://imagej.nih.gov/ij/	RRID:SCR_003070
Prism 6.0	GraphPad Software	N/A
Adobe Illustrator CS6	Adobe	N/A
dfRTPA	In house written Python script	N/A
MC_Stimulus II	Multichannel systems	N/A
Vortran PC	Vortran Laser Technology	N/A
nVista	Inscopix	N/A
Mosaic	Inscopix	N/A
Ethovision XT with external control module	Noldus	N/A

Genotyping

PCR amplification of extracted genomic DNA was used to determine the genotype of the animals. 1 µl of genomic DNA was used as the template together with 0.25 µM primers in a commercial PCR reagent mixture (Reddymix PCR Master Mix, ThermoScientific). The presence of the SF1-Cre gene was detected by the resultant 250 bp PCR product using previously described pair of primers (Dhillon et al., 2006): forward 5' – CTGAGCTGCAGCGCAGGGACAT – 3' and reverse 5' – TGCGAACCTCATCACTCGTTGCAT – 3'. The reactions were carried out in a 10 µl final volume in a thermocycler (C100, BioRad) under the following cycling conditions: 95 °C for 3 min, followed by 5 cycles of 95 °C for 1 min, 58 °C for 1 min, and 72 °C for 2 min, then a further 25 cycles of 95 °C for 1 min, 56 °C for 1 min and 72 °C for 1 min, and finally 72 °C for 5 min. *Z/eg*-positive genotype was confirmed with a forward 5' –AAGTTCATCTGCACCACCG – 3' and reverse 5' – TCCTTGAAGAAGATGGTGCG – 3' primer pair producing a 750 bp product, with the same cycling conditions used as for the SF1-Cre genotyping. The PCR products were separated by electrophoresis in a 3% agarose gel prepared with TAE buffer (40 mM Tris acetate pH 8.3, 1 mM EDTA) and 0.02% ethidium bromide, and were subsequently imaged by ultraviolet light illumination.

Assessment of competing appetitive and avoidance-inducing drives

Customised assays were used to evaluate the influence of SF1 neurons on the conflicts between appetitive and avoidance motivations by providing a choice between safe but food-lacking or potentially dangerous and anxiogenic but food-containing environments. *Ad lib* fed animals were tested for 3 hours in open field and 4-chamber assays, where open, illuminated space and innate fear-inducing olfactory (2,4,5-trimethylthiazole or TMT, Sigma Aldrich) cues were used to create avoidance-inducing environments respectively (Genné-Bacon et al., 2016). Overnight fasted animals were evaluated in a fear-conditioning box (Med Associates) that involved mild (0.4 mA) electric shocks for the simulation of a dangerous environment.

Real-time place avoidance assay

The real-time place avoidance (RTPA) assay was performed in 2-chamber arena as described previously (Kunwar et al., 2015). After an initial 10 min exploration, blue light (488 nm, 20 Hz or 2 Hz tonic pulses of 1 ms) was delivered for 20 min when the mouse entered the pseudo-randomly designated stimulation chamber, followed by an additional 10 min of free-exploration. To quantify effects of varying stimulation frequencies, a dynamic frequency RTPA (dfRTPA) assay was used. After an initial 5 min, an in-house Python script initiated a 2 Hz photostimulation whenever the animals was in the designated chamber and increased stimulation frequency by 0.5 Hz every 5 min to a maximum of 6 Hz. Fed or overnight fasted SF1-ChR2 animals were tested in a crossover manner and preference of the animals. This task was further adjusted to evaluate pre-existing place preference by testing mice in a successive alleys (SA) arena, where the designated stimulation chamber was the least anxiogenic alley 1, or to analyse the competition between food seeking and SF1 stimulation-induced avoidance under different adiposity states. In the latter assay, empty pots were placed at the opposite ends of the 2-chamber RTPA arena, 1 in each of the chambers. Animals were calorie restricted (CR) to 85% of their initial bodyweight or fed either chow or high fat diet for 2 weeks. All groups were overnight fasted and after initial 5 min, food was placed in the pot of the stimulation chamber. After a further 5 min, a 2 Hz optogenetic stimulation protocol was initiated and proceeded to increase by 0.5 Hz every 5 min to 6 Hz as described for dfRTPA above.

Food intake

For fast-refeed studies, animals were fasted overnight from 17:00. For DREADD studies, animals were administered CNO 30 min before returning food (0.5 mg/kg for SF1-hM3Dq and 2.5 mg/kg for SF1-hM4Di animals). For optogenetic studies, mice were pre-stimulated for 5 min with continuous 1 ms, 2 Hz 488 nm laser light pulses and stimulation continued for 5 h. For food intake measurements in a fed state, animals were injected with CNO at 12:30. For studies in the BioDAQ system (Research Diets, Inc) CNO was delivered via IP injections at 12:30 and 18:30 and food intake, feeding bouts and time spent engaged with the food were recorded. Data was exported in 1 hour bins and analysed in Excel.

Body composition

For chronic CNO studies, body composition was determined using an EchoMRI™-500 analyser on the day that the treatment was started and then weekly for the duration of the studies. Measurements of fat

mass, lean mass, total and free water were acquired consistently between 8:00 and 14:00 with triple acquisition settings.

Assessment of locomotion

A circular open field (COF) arena, 40 cm diameter and 40 cm height, was used to assess spontaneous mobility. Movement velocity over a 10 min period was quantified 30 min and 5 h post-CNO-injection. To evaluate horizontal (X) and vertical (Z) locomotion in familiar environments, animals were housed in Comprehensive Lab Animal Monitoring System (CLAMS, Oxymax) for at least 3 days to ensure habituation. Just before the dark period at 19:00, CNO was administered into the drinking water and infrared beam brakes were quantified.

Assessment of anxiety

The elevated plus maze (EPM), successive alleys (SA), elevated zero maze (EZM) and light/dark box (LD) were used as a measure of anxiety-like exploratory behaviour, 30 min and/or 5 h after CNO administration (Bourin and Hascoët, 2003; Braun et al., 2011; Deacon, 2013; Pellow et al., 1985). Increased time spent in open and/or more brightly illuminated areas in these tests was considered as a mark of decreased anxiety-like behaviour.

Conditioned place preference

Conditioned place preference (CPP) was assessed in three chamber automated conditioned place preference boxes (Med Associates). After assessing pre-existing biases for one of the chambers (day 1), animals were randomly assigned to the chamber in which they would receive the CNO injection. Over the next 4 conditioning days, animals were injected with vehicle prior to confinement to one chamber in the morning and then injected with CNO and confined to the alternate chamber in the afternoon. On the 6th day, animals were placed in the centre chamber and allowed to freely explore the CPP box for 15 min. Time spent in the vehicle- and CNO-paired chambers was quantified.

Dissections for tissue collection

In order to collect tissues for the assays described below, mice were culled by cervical dislocation. Brains were collected and kept in 4% paraformaldehyde (PFA) overnight, before being transferred to 30% sucrose until no longer buoyant. Then brains were frozen on dry ice and maintained at -80 °C. Other tissues were frozen in liquid nitrogen immediately after harvesting and also kept at -80 °C.

Perfusion

Animals were overdosed with sodium pentobarbital (Euthatal) and intracardially perfused with 10-20 ml of sterile PBS'A' buffer, pH 7.2 and buffered 4% PFA. Brains were dissected and incubated overnight in 4% PFA, then transferred into 30% sucrose until no longer buoyant. The brains were then frozen in dry ice and kept at -80 °C.

Histology

For c-fos immunohistochemistry assessment of hM3Dq functionality, SF1-hM3Dq animals were injected with CNO or vehicle via the intraperitoneal (IP) route and perfused 90 min later. For functional assessment of optogenetic activation, SF1-ChR2 or SF1-eYFP animals were stimulated at 2 or 10 Hz for 90 min and perfused afterwards. For immunoreactivity detection, rabbit anti c-fos monoclonal antibodies (catalogue number: 2250 from Cell Signaling Technologies) were used in combination with chicken anti-rabbit IgG ((H+L), conjugated with Alexa Fluor 647, Molecular Probes, catalogue number: A-21443) secondary antibody. Firstly, coronal brain sections (35-100 µm thickness) were placed in meshed wells and washed in a bath of sterile PBS'A' buffer, pH 7.2, under gentle rocking. Then the sections were transferred to a blocking solution (1% chicken serum, 1 mg/ml bovine serum albumin (BSA) and 12.5% Triton-X) and incubated at room temperature (RT) for 1 h. Afterwards, sections were incubated in a 1:400 primary antibody solution, prepared in blocking mixture, for 1 h at RT and then overnight at 4 °C. The following day, sections were washed for 30 min and incubated in a 1:400 dilution of secondary antibody in the blocking solution for 2 h at RT. Then, sections were washed again for 30 min and mounted on slides using Shandon Immu-Mount (#9990402, Thermo Scientific) and imaged (TCS SP5, Leica). For detection of mCherry and/or eGFP brains were sectioned, mounted and imaged as described above.

References

Armbruster, B.N., Li, X., Pausch, M.H., Herlitze, S., and Roth, B.L. (2007). Evolving the lock to fit the key to create a family of G protein-coupled receptors potently activated by an inert ligand. Proc. Natl. Acad. Sci. *104*, 5163–5168.

Bourin, M., and Hascoët, M. (2003). The mouse light/dark box test. Eur. J. Pharmacol. *463*, 55–65.

Braun, A.A., Skelton, M.R., Vorhees, C. V, and Williams, M.T. (2011). Comparison of the elevated plus and elevated zero mazes in treated and untreated male Sprague-Dawley rats: effects of anxiolytic and anxiogenic agents. Pharmacol. Biochem. Behav. *97*, 406–415.

Deacon, R.M.J. (2013). The successive alleys test of anxiety in mice and rats. J. Vis. Exp.

Dhillon, H., Zigman, J.M., Ye, C., Lee, C.E., McGovern, R. a, Tang, V., Kenny, C.D., Christiansen, L.M., White, R.D., Edelstein, E. a, et al. (2006). Leptin directly activates SF1 neurons in the VMH, and this action by leptin is required for normal body-weight homeostasis. Neuron *49*, 191–203.

Genné-Bacon, E.A., Trinko, J.R., and DiLeone, R.J. (2016). Innate Fear-Induced Weight Regulation in the C57BL/6J Mouse. Front. Behav. Neurosci. *10*, 132.

Kunwar, P.S., Zelikowsky, M., Remedios, R., Cai, H., Yilmaz, M., Meister, M., and Anderson, D.J. (2015). Ventromedial hypothalamic neurons control a defensive emotion state. Elife *4*, 1–30.

Novak, A., Guo, C., Yang, W., Nagy, A., and Lobe, C.G. (2000). Z/EG, a double reporter mouse line that expresses enhanced green fluorescent protein upon Cre-mediated excision. Genesis *28*, 147–155.

Pellow, S., Chopin, P., File, S.E., and Briley, M. (1985). Validation of open : closed arm entries in an elevated plus-maze as a measure of anxiety in the rat. J. Neurosci. Methods *14*, 149–167.
